# Adaptation of central metabolite pools to variations in growth rate and cultivation conditions in *Saccharomyces cerevisiae*

**DOI:** 10.1186/s12934-021-01557-8

**Published:** 2021-03-09

**Authors:** Kanhaiya Kumar, Vishwesh Venkatraman, Per Bruheim

**Affiliations:** 1 Department of Biotechnology and Food Science, Norwegian University of Science and Technology (NTNU), 7491 Trondheim, Norway; 2 Department of Chemistry, Norwegian University of Science and Technology (NTNU), 7491 Trondheim, Norway

**Keywords:** Metabolomics, Bioreactor, Chemostat, Mass Spectrometry, Yeast, Glycolysis, Physiology

## Abstract

**Background:**

*Saccharomyces cerevisiae* is a well-known popular model system for basic biological studies and serves as a host organism for the heterologous production of commercially interesting small molecules and proteins. The central metabolism is at the core to provide building blocks and energy to support growth and survival in normal situations as well as during exogenous stresses and forced heterologous protein production. Here, we present a comprehensive study of intracellular central metabolite pool profiling when growing *S. cerevisiae* on different carbon sources in batch cultivations and at different growth rates in nutrient-limited glucose chemostats. The latest versions of absolute quantitative mass spectrometry-based metabolite profiling methodology were applied to cover glycolytic and pentose phosphate pathway metabolites, tricarboxylic acid cycle (TCA), complete amino acid, and deoxy-/nucleoside phosphate pools.

**Results:**

Glutamate, glutamine, alanine, and citrate were the four most abundant metabolites for most conditions tested. The amino acid is the dominant metabolite class even though a marked relative reduction compared to the other metabolite classes was observed for nitrogen and phosphate limited chemostats. Interestingly, glycolytic and pentose phosphate pathway (PPP) metabolites display the largest variation among the cultivation conditions while the nucleoside phosphate pools are more stable and vary within a closer concentration window. The overall trends for glucose and nitrogen-limited chemostats were increased metabolite pools with the increasing growth rate. Next, comparing the chosen chemostat reference growth rate (0.12 h^−1^, approximate one-fourth of maximal unlimited growth rate) illuminates an interesting pattern: almost all pools are lower in nitrogen and phosphate limited conditions compared to glucose limitation, except for the TCA metabolites citrate, isocitrate and α-ketoglutarate.

**Conclusions:**

This study provides new knowledge-how the central metabolism is adapting to various cultivations conditions and growth rates which is essential for expanding our understanding of cellular metabolism and the development of improved phenotypes in metabolic engineering.

**Supplementary Information:**

The online version contains supplementary material available at 10.1186/s12934-021-01557-8.

## Background

*Saccharomyces cerevisiae* is a well-known popular model system for basic biological studies. It is also of considerable industrial interest, ranging from traditional bioprocesses of beer and wine production to serve as a host organism for heterologous production of commercially interesting small molecules and proteins. *S. cerevisiae* is a respiro-fermentative microorganism with the characteristics of having the long-term Crabtree positive effect, which can produce ethanol under aerobic conditions when fed with a high glucose concentration [1]. A high level of glucose represses the tricarboxylic acid (TCA) cycle and respiration, and pyruvate can overflow to ethanol through the high capacity pyruvate decarboxylase and constricted flux through pyruvate dehydrogenase [2]. Thus, this species can maintain active metabolism and growth under many different cultivation conditions. In this report, we explore how the central metabolite pools of *S. cerevisiae* adjust to the growth rate and cultivation conditions, a topic that has only been partly addressed in the literature.

The analysis of the metabolome–metabolomics–has gone through a remarkable instrumental and methodological development during the last two decades. NMR and mass spectrometry (MS) are the two main detection technologies used, the latter with superior properties for highly sensitive and comprehensive coverage of the metabolome. The MS-metabolomics workflow still faces major challenges in all steps, especially high recovery sampling and sample processing is a long-standing non-resolved topic. Besides, the mass spectrometer is a challenging detector when used for absolute quantification purposes [3]. Regardless, high-quality metabolome studies are continuously reported, adding new knowledge to biological function and mechanisms but are also used for guidance and evaluation in metabolic engineering projects [4–6]. Most studies are designed and interpreted at a relative scale, i.e. mutant vs. wild-type/ reference strains, but the ultimate goal is to report absolute intracellular concentrations [7]. Such information is essential to testing and validating kinetic models [8], to increase understanding in genotype–phenotype interactions and cellular engineering [9–11]. These data will be important for further emphasis to integrate different level omics-data with mathematical models, especially genome-scale metabolic models, of biological systems.

The available metabolome databases (e.g. *E. coli*/ Yeast, Human metabolome databases) contain an impressive collection of metabolite data but considerably lower amounts of information on intracellular concentrations [12–14]. Importantly, concentration entries can range several orders of magnitude for some metabolites. This may be attributed to the fact that different methodologies have been applied by different labs and are also under continuous development. Furthermore, sampling is often performed on cells grown under different conditions and in different physiological states. The metabolomics community has undertaken efforts to standardize metabolomics workflows [15] and an initiative to select a few model organisms to advance the field of Metabolomics [16].

Our group has developed a set of quantitative LC–MS/MS methods for central metabolism, all methods using [13] C-Isotope dilution strategy for the highest level of quantitative precision and accuracy [17–22]. The core metabolism is centered on the glycolytic and pentose pathways, TCA, and energy-conserving mechanisms. Important metabolite classes are sugar phosphates and other phosphorylated metabolites, TCA organic acids and pyruvate and excreted metabolites like ethanol, lactic and acetic acids, amino acids, nucleoside mono-/di-/tri-phosphates. These pathways and metabolites not only serve to make energy available but also act as a precursor for the macromolecular synthesis and are particularly interesting to monitor. We have applied this methodology in a previous study to report the absolute intracellular concentration of several popular microbial and mammalian model systems, using the early exponential growth phase as the reference physiological state [23].

Now, we turn the focus on central metabolite pools in baker’s yeast and how the composition varies with growth rate and cultivation condition. It has been known for many decades that macromolecular composition varies with growth rate [24], but there is limited information on how central metabolite pools are adjusted with the growth rate. For our study, we chose to use the frequently investigated *S. cerevisiae* cen.pk strain [25, 26]. Boer and co-workers ran a series of metabolite profiling of yeast at steady-state in chemostats and varied both growth rate and nutrient limitations [27]. They reported a strong correlation in responses on metabolite concentration to nutrient limitations. Christen and Sauer performed a combined metabolome and fluxome study on seven yeast species, including *S. cerevisiae* [2]. They found the metabolite pool compositions to be mainly species-specific, but, interestingly, an overarching-species metabolic flux correlation for fructose-1,6-diphosphate and dihydroxyacetone-phosphate was found. Most metabolome studies on yeast do not report absolute concentration and focus on using metabolite profiling in strain development and stress-situations, e.g. de Ruijer and co-workers used a quantitative metabolomics approach for study the metabolic burden of recombinant antibody production in *S. cerevisiae* [9], Nishino and co-workers investigated *S. cerevisiae* strains lacking PFK1 and ZWF1 using absolute quantitative methodology [28], Jung and co-workers used intracellular metabolite profiling for characterization of adaptation of *S. cerevisiae* to furfural stress [29]. These high-quality studies provide interesting information on how yeast cells adapt to genetic changes and stress exposure. One challenge for such studies is how to interpret the metabolite data if a mutant strain is growing at a different growth rate than the wild-type and if the stress exposure causes changes in growth rates. It might be that the resulting metabolite profiles are more consequences of the adaptation to changes of growth rate rather than the direct effect of the stress. A deeper insight into cellular adaptation at the central metabolome level to growth rate is highly needed, from a basic scientific point of view but also as a guide for interpretation of other Metabolome studies. The substrate consumption rate is also pointed to as a core variable to control the metabolic phenotype [30]. This is the background and motivation to undertake the present study in the model organism *S. cerevisiae*. Both batch (exponential and stationary phase) and chemostats (glucose, nitrogen, and phosphate limited) at a series of dilution rates i.e. growth rates, were included since both bioreactor modes of operation are relevant in yeast physiology studies. We included also high dilution rates of the chemostats since we wanted to study the transition from nutrient limitation to unlimited growth. We envision that more studies, both inter-/ intra-species and with variable cultivation conditions including various stress testing should follow.

## Results

The outline of the Results section is first to present the cultivation data as they are important constraints on the evaluation and interpretation of the intracellular metabolite pool data. This is followed by the presentation of the absolute concentration of all individual metabolites across all sampling conditions and next pooled into the respective metabolite groups. Further, a series of multivariate analyses were done, both on complete data set and within one-variable conditions. Finally, more closer inspections with pairwise comparisons are performed.

### Cultivation data

**Fig. 1:**
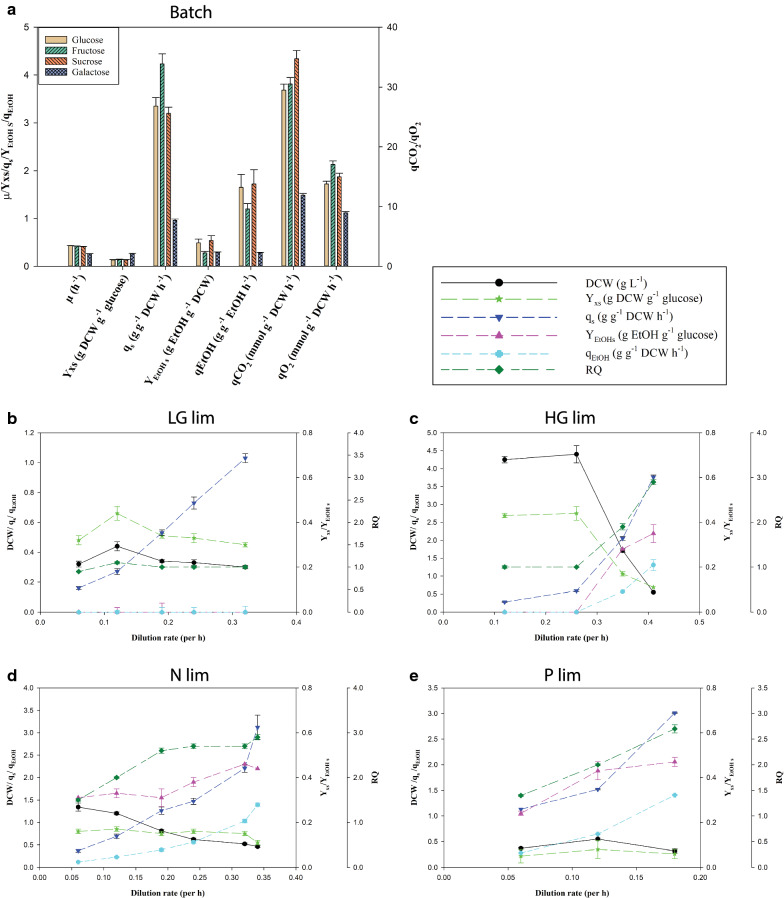
Offline and online cultivation data for Batch (**a**), Low Glucose limited chemostat (**b**), High Glucose limited chemostat (**c**), Nitrogen limited chemostat (**d**), and Phosphate limited chemostat (**e**). Units for the Yield (Y) and specific rates (q) are given in the text box. RQ is the respiratory coefficient (ratio of CO_2_ production and O_2_ consumption)

*Batch cultivation on glucose, fructose, galactose, and sucrose.* The growth performance of *S. cerevisiae* CEN.PK on glucose, fructose, and sucrose (GluFruSuc) were nearly similar as indicated by different growth parameters such as biomass yield on substrate, specific growth rate, specific CO_2_ evolution rate, specific substrate uptake rate (Fig. 1a, tabulated data can be found in Additional file 1: Table S1). The exponential phase lasted 7 to 8 h on all three carbon sources using the traditional yeast mineral medium with 15 g carbon source L^−1^ (see Additional file 2: Figure S1 for online CO_2_ and O_2_ offline gas profiles) introduced by Verduyn and co-workers [31]. The specific growth rates (µ) on glucose, fructose, and sucrose were nearly similar having values 0.43 h^−1^, 0.42 h^−1^, 0.41 h^−1^, respectively. Sucrose was hydrolyzed into glucose and fructose during the cultivation, and it was not detected in the broth after three hours of cultivation. Like several previous reports, glucose was preferentially consumed over fructose, which led to an initial build-up of fructose and the presence of fructose in the broth even after consumption of glucose. This may be the reason for a nearly similar growth performance on sucrose as compared to fructose and glucose. Our result is corroborated with previous studies on this strain [32–34], e.g. van Dijken and co-workers reported µ of 0.41 h^−1^ and biomass yield of 0.12 g biomass g^−1^ on glucose and µ of 0.38 h^−1^ on sucrose in shake flasks [25]. The growth performance on galactose was contrasted by a 13 h long exponential phase, lower specific maximum growth rate of 0.26 h^−1^, low galactose uptake rate, the low release of fermentative products as ethanol, low CO_2_ evolution, and O_2_ consumption rate compared to GluFruSuc (Fig. 1a). However, the biomass yield was 0.26 g g^−1^ DCW after the exponential phase, which was nearly two-fold higher compared to biomass yield after the exponential phase on GluFruSuc. This growth performance is similar to the previously reported [35]. *S. cerevisiae* CEN.PK is a Crabtree-positive strain and trace amounts of acetic acid, glycerol, succinic acid, and α-ketoglutarate (neither reported) were also detected in addition to ethanol. The exponential aerobic respiratory-fermentative growth phase on sugars was succeeded with re-uptake and catabolism of ethanol as shown in earlier studies on glucose [25]. Metabolite profiling was performed in this phase also for three sugars (GluFruSuc).

*Chemostats* are strict nutrient-limited over the range of dilution rates with constant biomass yield [36]. For this study, we also chose to increase the dilution rate above this threshold and approaching the maximum growth rate,and wash out situation. Extracellular and metabolome samplings were performed after four to six volume exchanges where all monitoring parameters were stable (except highest dilution rate for phosphate limited).

The carbon (glucose) limited chemostat was operated in two different inlet glucose concentrations: low glucose (LG, feed glucose concentration of 1 g L^−1^) and high glucose (HG, feed glucose concentration of 10 g L^−1^) but still the limiting nutrient (Fig. 1b, c, tabulated data can be found in Additional file 1: Tables S2–3). The highest tested dilution rate (0.41 h^−1^) on HG chemostat approaches maximum specific growth rate but washout was not observed. Biomass yields were constant and at maximum for the two lowest dilution rates (0.12 and 0.24 h^−1^) and by-product formation was negligible). The respiratory quotient (RQ) was close to one which indicates fully respiratory growth. This was corroborated with previous results where a decrease in glucose uptake rates were found to decrease in the secretion of the main fermentative product ethanol [2, 25, 37]. The threshold for the onset of the Crabtree effect seems to be between specific growth rates of 0.26 h^−1^ and 0.35 h^−1^ and approaching the same ethanol yields (Y_EtOHS_) as the unlimited batch cultivation on glucose. The threshold specific growth rate can be defined as a specific growth rate where yeast starts to show respiro-fermentative growth. The Crabtree effect is usually discussed as a sugar concentration-dependent effect and induced at higher concentrations, but in the HG chemostat at µ = 0.35 h^−1^, most of the glucose was consumed, indicating that higher growth rates/ glucose consumption rates trigger this overflow mechanism also and not only high sugar concentrations. An increased specific glucose uptake rate enhances the glycolytic flux, which results in the diversion of pyruvate to both directions: TCA cycle and fermentative routes. This was also indicated by a high RQ at higher dilution rates. The RQ value higher than one indicates the diversion of pyruvate towards fermentative routes where one mole of pyruvate is consumed to produce one mole of carbon dioxide and one mole of ethanol without consuming oxygen. The appearance of ethanol was reported to be the most sensitive indicator for the onset of respiro-fermentative metabolism (van Dijken et al. [25]). In any case, this series of growth rates are interesting to study at the intracellular metabolite pool levels to monitor adaptation from fully aerobic to respiro-fermentative metabolism. Growth parameters of LG chemostats were the same as HG chemostats for comparable dilution rates (Additional file 1: Tables S2, 3). Ethanol was not detected at the highest dilution rate 0.31 h^−1^ which indicates that onset of the Crabtree effect is close to 0.35 h^−1^ in glucose-limited chemostats, while ethanol production was observed in galactose batch cultivation at µ = 0.26 h^−1^.

The nitrogen (ammonium) limited chemostat was operated in the range of dilution rates of 0.06 to 0.34 h^−1^ and contrary to carbon limitation, the entire growth rate range was respiro-fermentative (Fig. 1d, tabulated data can be found in Additional file 1: Table S4**)**. The steady-state biomass and ethanol concentration had a decreasing trend with an increase in the dilution rate while the glucose concentration in the outlet was increased with the dilution rate. However, on a specific rate basis, there is an increased ethanol yield. Also, the increase in dilution rate led to the enhancement of specific respiration rate and RQ. The contribution of CO_2_ in the carbon recovery was increased at higher dilution rates while the carbon recovery was mostly dominated by biomass and ethanol production at lower dilution rates. The specific glucose uptake rate was higher in nitrogen than glucose-limited chemostats at the same dilution rates which indicates a higher catabolic activity of glucose when this nutrient is not limited.

The phosphorous (phosphate) limited chemostat was operated at the dilution rate of 0.06 h^−1^, 0.12 h^−1^, 0.18 h^−1^. However, a steady-state off-gas composition was not observed at 0.18 h^−1^ after 4–6 volume exchanges though the culture was almost stable in terms of optical density. Like nitrogen-limited chemostat, the phosphate-limited chemostat had a higher specific glucose uptake rate, RQ, and excretion of fermentative products in comparison to the glucose-limited chemostat (Fig. 1e, tabulated data can be found in Additional file 1: Table S5). Ethanol production was also observed at all dilution rates, and, interestingly, a higher specific glucose consumption rate and ethanol yield were observed for the phosphate limited vs. nitrogen and glucose at the same growth rate (0.12 h^−1^).

In all, our data reproduces previous reports where similar conditions were tested, and besides, we included conditions where chemostat dilution rates approach maximum growth rate. A plot of substrate consumption rate vs. growth rate shows a quite linear correlation for all conditions, except for the high dilution glucose limited chemostat that trigger ethanol formation (Additional file 2: Figure S12). All these extracellular substrate consumption and production formation rates are important data for the interpretation of the endometabolome data presented next.

### Quantitative profiling of intracellular metabolite pools

**Fig. 2:**
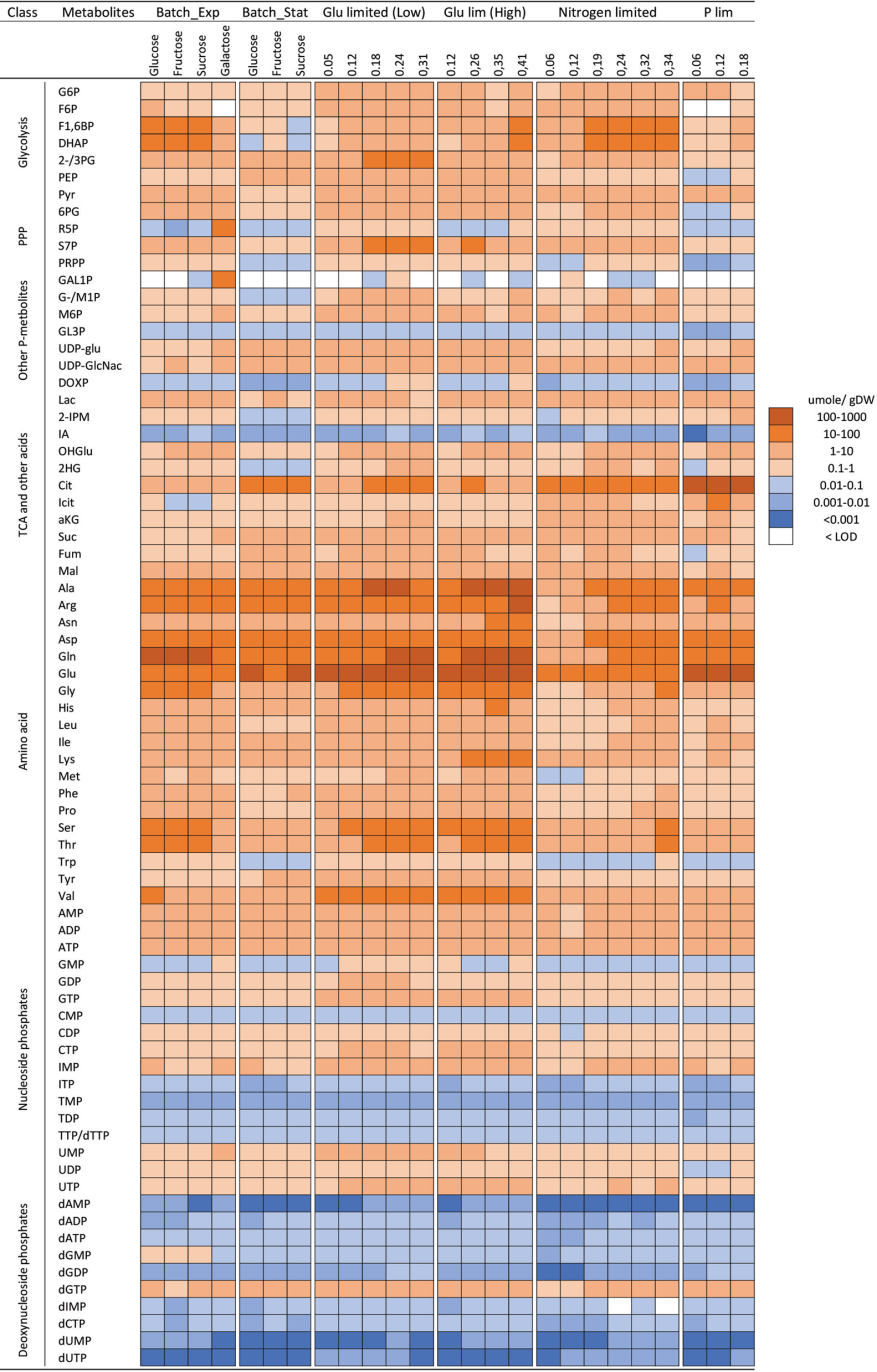
Heat-map showing the abundance of individual intracellular metabolite concentration represented in logarithmic scale across different cultivation conditions in batch and chemostat

**Fig. 3:**
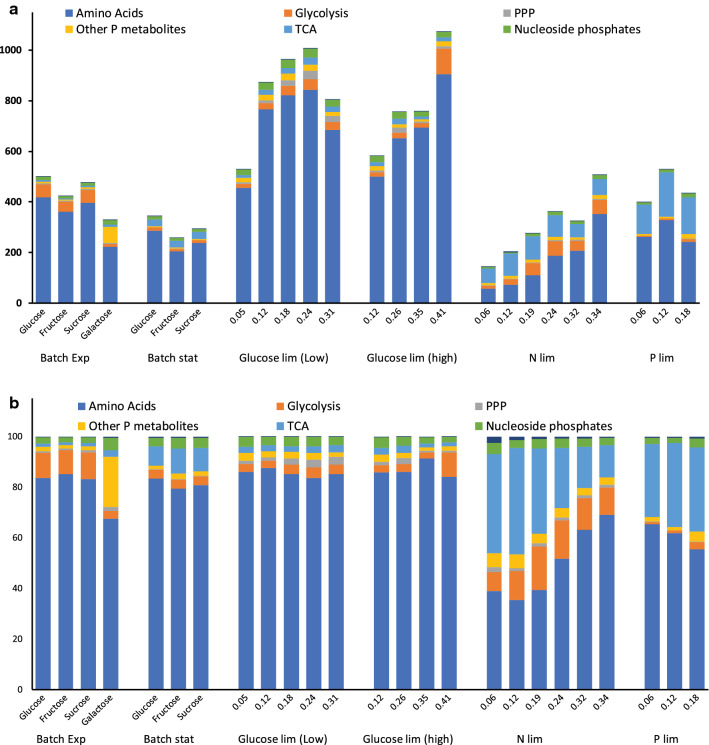
The upper bar diagram shows the abundance of the group of intracellular metabolites concentration and the lower bar diagram shows the relative fraction of metabolites pools represented in terms of percentage

*Absolute intracellular concentration* (in µmole/ g DW units) of all quantified metabolites across different cultivation conditions are shown in a logarithmically scaled heat-map (Fig. 2, numbers are given in Additional file 3: Table S6) with corresponding relative standard deviations enclosed in Additional file 2: Figure S2 (also in numbers in Additional file 3: Table S6). At first glance, the overall picture indicates a large degree of similarities in the metabolite pools, i.e. high abundant amino acids, low abundant deoxy -nucleotides, but when summarizing individual metabolite pools into respective metabolite classes a relatively large variation is detected (Fig. 3). First, there is a quite large difference in total metabolite pools with the highest in glucose-limited chemostats (Fig. 3, upper panel), and on a fractional scale the phosphate and nitrogen-limited chemostats stand out with relatively lower amino acid pools and higher TCA pools (Fig. 3, lower panel). There is also a trend with increased total pools with the growth rate for glucose and nitrogen-limited chemostats. Closing in at the individual molecule level is, as expected, a large range of concentrations observed (Additional file 2: Figure S3, left panel) as earlier indicated in the heatmap of Fig. 2. Glutamate, glutamine, alanine, and citrate are the four most abundant metabolites. Interestingly, glycolytic and PPP metabolites display the largest variation among the cultivation conditions while the nucleoside phosphate pools display the least variations (Additional file 2: Figure S3, right panel). At this coarse level of interpretation, there is no inconspicuous correlation between different batch and chemostat cultivations nor Crabtree-effect, i.e. ethanol-producing cultivation conditions (all batch, all N- and P-limited chemostats and high dilutions rate C-limited chemostats) and overall metabolite pool composition.

*Multivariate analysis.* Submitting the total data set to PCA revealed a clear clustering of glucose-limited chemostats at all dilution rates vs. the other conditions along PC1 of the scores plot, while GluFruSuc batch cultivations are separated from N-/P lim chemostats along PC2 (Additional file 2: Figure S4, upper panel). Interestingly, the galactose batch is closer to N-/P lim chemostats than the GluFruSuc batch conditions. There is also a general trend that PC2 separates on growth rate and all glucose-limited chemostats operating at non-Crabtree conditions (i.e. no ethanol production) are collected in the lower right quadrant of the scores plot. This view is supported by the loadings plots (Additional file 2: Figure S4, lower panel) where the growth, substrate, and ethanol rates naturally contribute along PC2. Metabolites contributing to the PC1 dimension are scattered among the metabolite classes and no pattern is apparent at this level. However, it is worth noting that ATP, ADP, and AMP, and G6P are not correlated to growth (orthogonal in loadings plot), and glutamate being a central metabolite in nitrogen metabolism is almost inversely correlated to substrate consumption and ethanol production.

Spearman rank correlation analysis is more suitable for the identification of any potential general patterns, with a particular focus on the correlation between the extracellular ratesand intracellular data, the former being fluxes and the latter pool sizes. The analysis was performed on complete data set and localized to the individual five cultivation conditions (1 batch, 4 chemostats). It is rather naïve to expect the correlation between the extracellular rates (growth, substrate, and ethanol production) and the global data set levels, and no apparent trends were observed (Additional file 2: Figure S5), neither much on the individual cultivation conditions either (Additional file 2: Figures S6–10), maybe except the observation in N-limited chemostats that citrate and isocitrate are negatively correlated with most other metabolites while α-ketoglutarate, being next in TCA, is slightly positively correlated to many metabolites (Additional file 2: Figure S9). However, one interesting pattern is that there is little/no correlation within members of the same metabolite class, rather individual metabolites have no apparent correlation within class nor pathways but are scattered along the axis on the correlation plots. This contrasts with the noticeable difference in metabolite class variations seen in Additional file 2: Figure S3, right panel.

Next, a more directed correlation analysis was performed between the carbon source consumption rate and the other rates (growth, O_2_, CO_2_, ethanol) and the intracellular metabolite pools since it has been suggested that the substrate rate control the global metabolic phenotype [30]. This was performed by single pairwise regression analysis and inspection of slopes and r-square values for the total data set, and each of individual N lim chemostats, Glim chemostats, ethanol-producing conditions, and non-ethanol producing conditions (Additional file 6: Table S9). All these different tests are necessary since in this study there are multiple variables (unlimited vs. limited growth, various growth rates, and carbon sources), which support respiration only and respiratory-fermentative phenotype. As expected, there is a strong correlation between the substrate consumption and the other rates (ethanol and CO_2_ production and O_2_ consumption) for most of the tests, confirming the summary of many studies performed by Huberts and co-workers with a particular focus on the correlation between ethanol production and substrate consumption [30]. However, no unique metabolite(s) stands out with a strong correlation to increased substrate consumption when all cultivation conditions are included. The correlation of most of the metabolites varies considerably among the tests, which is not surprising and underlines the flexible adaption to various external conditions by the central metabolism. Several metabolites, eg. F1,6BP, αKG, Gln are identified to play key roles in the regulation of the cell state [38]; thus, they should vary in a manner reflecting the changing conditions that provoke an intracellular response, i.e. either to coordinate gene expression or direct regulation of metabolic fluxes by enzyme modifications or interactions [22, 39, 40]. Here, one can conclude that neither the three mentioned metabolites nor the others co-vary solely with the substrate consumption, but other environmental conditions and cellular mechanisms matter. F1,6BP is still the metabolite with the highest degree of correlation, possibly together with the neighboring DHAP. αKG is not correlating with the substrate consumption in N lim chemostats but shows only a strong correlation in the respiratory Glim chemostats, where there is no ethanol production. Gln, on the other hand, is correlating with substrate consumption under Nlim conditions, and also Glim with and without ethanol production. This correlation analysis was redone on a data set normalized to total metabolite pools since the total metabolite pools vary quite significantly among the cultivation conditions (Fig. 3a), but no obvious new patterns emerge during this data processing (Additional file 6: Table S9.) No potential outliers were excluded in this first round of data inspection, but such trimming of the data could potentially reveal stronger correlation patterns.

**Fig. 4:**
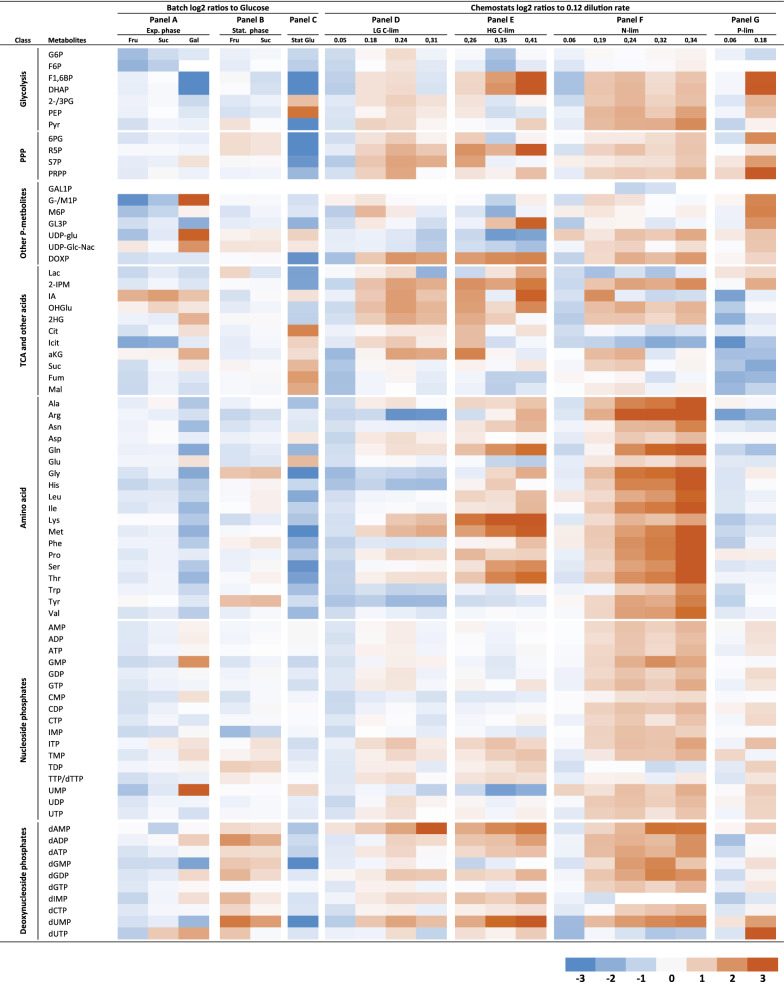
Panels A to G shows relative metabolite concentrations at log2 scale vs. glucose batch cultivation and dilution rate of 0.12 h^−1^ for chemostat cultivations (for individual nutrient limitations). Max values for color formatting are set to -3 and 3

*Pairwise comparisons.* Next, pairwise log2 comparisons were performed to several selected reference conditions: Glucose batch exponential, Glucose batch stationary, LG/HG/ Nlim/ Plim chemostats at 0.12 h^−1^ dilution rate (Fig. 4). Statistical analysis (Two-tailed t-test) for all pairwise comparisons in Panel A–E can be found in Additional file 5: Table S8. The percentage of significantly changed metabolites (p < 0.05) is between 50–70% for almost all comparisons, although there were no obvious patterns among the conditions being discovered from this test. However, there are two outliers (Sucrose vs glucose batch exponential growth, and N-lim 0.06 vs 0.12 dilution rates) with less significantly changed metabolites (32 and 23%, respectively).

The metabolites pool composition of fructose and sucrose cultivations are not that different from the glucose batch cultivation (Fig. 4a for exponential cultures and Panel B for stationary phase/ ethanol consuming conditions). Interestingly, G6P, F6P, and Pyr pools are quite lower in the fructose cultivation having a higher substrate uptake rate and lower ethanol production rate. As expected, most of the yeast metabolome on sucrose was in between the range of yeast metabolome on fructose and glucose. However, growth on galactose leads to a different composition of all metabolite classes of the central metabolome. If this is due to the catabolism of galactose directly or indirectly due the 40% reduction in growth rate can’t be separated in unlimited batch growth cultivations. The metabolite pools involved in the Leloir pathway for galactose assimilation were enhanced several folds, while the whole amino acid pool is downregulated (Fig. 4a and Additional file 2: Figure S11). The latter can be a consequence of the lower growth rate as the amino acid pools also went down in the ethanol-consuming stationary phase of the glucose batch cultivation (Fig. 4c, stationary vs exponential glucose batch cultivations). Further for the galactose cultivation; among nucleoside phosphates, all monophosphates were high in concentration. UDP-glu/gal, UDP-Glc/Gal-NAc were nearly 5 and threefold higher compared to GluFruSuc cultivated. Intermediates in the lower part of glycolysis were down-regulated while PPP metabolites and especially upper glycolytic intermediates were up-regulated several times in comparison to GluFruSuc cultivated yeast. DHAP and F16BP were several folds lower in comparison to GluFruSuc cultivated yeast. Pools of the TCA metabolites citrate, isocitrate, and αKG were higher. Interestingly, all metabolite groups except TCA metabolites went down in the ethanol consuming phase, the latter pools increased 2–4 times (Fig. 4c).

The overall trends for glucose and nitrogen-limited chemostats are increased metabolite pools with increasing growth rate (Fig. 4d–f). In both LG and HG, chemostats are most glycolytic, PPP, and TCA pools increasing. One difference between high and low glucose is the much larger upregulation of amino acid pools with the growth rate in the HG chemostats (Fig. 4d vs e). The HG carbon limited chemostat was characterized by a significant reduction in glutamate and the opposite trend with glutamine. This resulted in glutamine as the most dominant amino acid which is like the case of batch culture at the exponential phase (Fig. 2, Additional file 3: Table S6). Therefore, glutamine concentration can be viewed as a signature metabolite showing an abundance of carbon sources (glucose or fructose) in the medium.

The increase of amino acid pools is even more prominent for the nitrogen-limited chemostat series (Fig. 4f). There is also a significant increase in glycolytic and PPP metabolites. Boer and co-workers (2010) reported that glycolytic intermediates adjust to meet growth requirements and generally have the tendency to increase with increased glucose uptake which is confirmed in our study. Interestingly, increased amino acid pools with growth rates are not observed for the phosphate limited chemostats (Fig. 4g), rather the sharp increase in glycolytic and other phosphometabolite pools and decrease of TCA metabolite pools are the most prominent findings, but note that only low and medium dilution rates were studied under phosphate limited conditions.

**Fig. 5:**
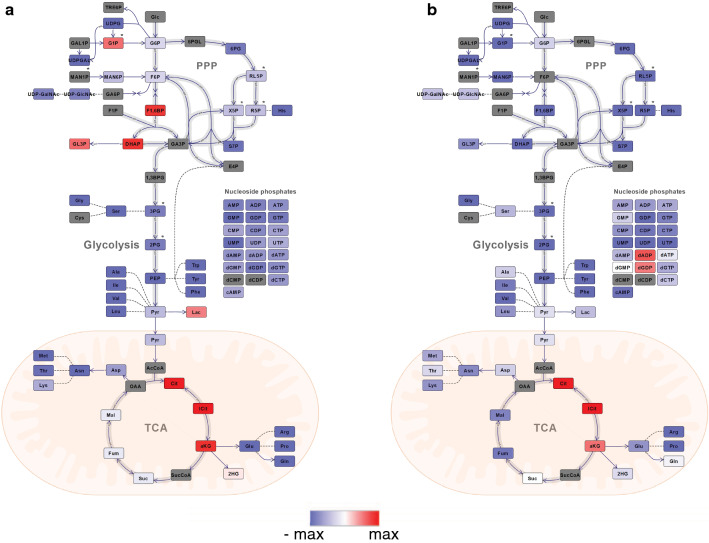
A plot showing changes in the metabolites of central metabolic pathways. This plot is plotted based on logarithmic value (base 2) of the ratio of intracellular metabolites concentration of *S.cerevisiae* in (**a**) nitrogen to glucose and (**b**) phosphorous to glucose-limited chemostat at the dilution rate of 0.12 h^−1^. Heatmap coding is from–max (blue) to max (red), below LOQ/uncertain/ not detected metabolites are grey

Finally, comparing the chosen reference growth rate (0.12 h^−1^) for the three different nutrient chemostat limitations illuminates an interesting pattern: almost all pools are lower in nitrogen and phosphate limited conditions, except for the TCA metabolites citrate, isocitrate, and α-ketoglutarate (Fig. 5a, b). F16BP and DHAP are upregulated for the nitrogen-limited condition only.

## Discussion

This study provides the most comprehensive collection of central metabolite concentrations over multiple cultivation conditions in *S. cerevisiae*. The findings sometimes confirm previous reports where similar cultivation conditions have been studied, but and not surprisingly there are also some results that point in different directions, e.g. we find amino acid pool reduction in nitrogen and phosphate limited chemostats compared to glucose-limited, while it was previously reported that glucose and nitrogen limitation were characterized by low amino acids pools and not phosphate limited chemostats [27]. There are many variables in a biological study, from lab strain differences, medium composition, and cultivation conditions, e.g. in bioreactors can pH and oxygenation be controlled but not in shake flasks, that contribute to differences in reports from various laboratories. Another major reason is the analytical approach. Most previous studies report relative values or quantitation based on a few standards and GC–MS has been as frequently used as LC–MS although LC–MS gradually seems to become more dominant. Thus, there are important initiatives to establish more standardized metabolomics workflows and selection of model organisms [16]. Here, we employ three LC–MS methods and all analytes are quantified using individual standard curves and ^13^C-labeled internal standards (either commercially available or application of ^13^C-glucose yeast extract). Mass spectrometry detection is concentration-dependent (the ionization step) and correction with ^13^C internal standards dramatically increases the precision of the quantitation. The accuracy of the ^13^C-Internal standard dilution strategy for LC–MS analysis and highest level reproducible microbial cultivation in bioreactors is at the 10–30% relative deviation among technical replicas (resampling from the same culture) and 10–20% total among biological replicas (independent cultivations), implying that more variation is introduced during sampling and sample processing and not among independent cultivations for single-cell microbial model system [23]. Thus, in total, we can discuss true biological changes at the levels of 30–50% increase and decrease in pools, even for many of the low abundant metabolites, that usually are reported with higher variations. This also justifies our revisit to previously reported yeast cultivation conditions, although this study was also expanded with more cultivation conditions and better coverage of central metabolite pools. The quantitative Metabolomics methodology has now matured to an advanced level and opening for comprehensive studies of metabolic responses to e.g. stress exposures, gene knock in/out, etc. at the absolute concentration reporting, which also is promising for further discovery of how metabolites contribute to sense and regulate metabolic flux and physiology in general [30, 38, 41].

Metabolites have different functions, from precursor metabolites in central metabolism via intermediates to building blocks in macromolecular assembly, some metabolites are important signaling molecules (e.g. cAMP, ppGppp), butyrolactones), others important energy (ATP) and redox carriers (NAD(P)H), and, importantly, some are present only at local nodes while others have many interactions/ roles in the global metabolic network. While some metabolites are known to contribute in the regulation of enzyme activity and gene expression [38–40], recent original and opinion publications discuss how metabolites and metabolite levels contribute in metabolic flux-sensing and further how metabolic flux can regulate physiological state, although latter mechanisms are far from resolved and understood but more point to focus in future experimentation [41]. All these aspects contribute to the challenge of how to interpret metabolome data, e.g. metabolite pools can both increase and decrease by increased turnover rates (i.e. flux), but, in any case, any change in metabolite levels indicate perturbation around that specific node/ pathway. The growth rate has for many decades been known as one main determinant for the macromolecular composition of microbial cells [24], but recent data indicate that the substrate uptake rate is also important for the metabolic phenotype including the metabolome composition. Thus, these two rates were central guides in the data evaluation and interpretation of this study. Energy charge ratio (ECR -the relationship among ATP, ADP, and AMP, range between 0 and 1 [42]) was consistent and about 0.6 across different types of nutrient limitations and specific growth rates (Additional file 4: Table S7). Thus, a stable energetic state is probably a priority for the cell. Another trend in the global data set is the higher relative variation in glycolytic and PPP pools vs amino and organic acids, and nucleoside phosphate pools being the less variant among all the cultivation conditions (Additional file 2: Figure S3). This data presentation also shows that F1,6BP is the metabolite with the relative largest variation, which is a prerequisite for a regulatory metabolite in addition to the needed positive correlation with other variables, growth, and substrate consumptions rates being the most important. It is hard to point to other high variable metabolites with potential regulatory roles in the same plot, and the well-known regulatory metabolite αKG is among the least variable in this plot.

However, more patterns/ observations emerge during the inspection of sub-sets of cultivation conditions F1,6BP and DHAP were observed in Crabtree-positive yeasts like our *S. cerevisiae* cen.pk strain to span in the range of tenfold increase or decrease in concentration which led to the suggestion that these metabolites can potentially function as a general flux indicator of glycolysis and ethanol secretion [2]. These two metabolites also showed the strongest correlation with ethanol production among our panel of cultivation conditions and metabolite coverage (Additional file 2: Figure S5). In this regard, it is also interesting to note that F1,6BP and DHAP were significantly upregulated in nitrogen vs. glucose-limited chemostats (Fig. 5a). Glutamic acid is well known to play a central role in nitrogen metabolism because of its dominance (first or second place) in the total amino acids pool irrespective of cultivation conditions, types, and severity of nutrient limitation as shown in the present study and many previous [43]. α-ketoglutarate, placed at the intersection of carbon and nitrogen metabolic pathways, has also emerged as a key master regulatory metabolite [39] and not only for regulation of carbon metabolism but also nitrogen metabolism [38]. This is also illuminated in the current metabolite profiling data set when comparing nitrogen vs. glucose-limited chemostats (Fig. 5a). It is also interesting to note is the sharp accumulation of the three first TCA acids incl α-ketoglutarate in the phosphate limited vs glucose-limited chemostat not being reported before to our knowledge (Fig. 5b). However, the cultivation data must be included in the interpretation since glucose consumption is almost five times higher in phosphate limited chemostat that also shunts 40% of the glucose carbon to ethanol while there is no ethanol production in the glucose-limited chemostat. Biomass yield is however four times higher in the glucose-limited chemostat. Altogether this indicates a large re-distribution in the intracellular metabolic fluxes which also is manifested by down-regulation of all metabolite pools except citrate, isocitrate, and a-ketoglutarate at the same growth rate of phosphate limited vs glucose-limited chemostats.

A deeper evaluation of the results, e.g. by kinetic model and simulations strictly requires enzyme concentrations [8, 44], but various computational approaches have been developed [5]. Experimental determination of main intracellular fluxes using ^13^C-label studies are also highly desired for validation/ can be used as constraints on modeling and simulation. Such multi-omics studies are extremely resource-demanding, but this study shows that the analytical metabolite profiling methodologies are ready for such future studies. In all, we have presented the most comprehensive central metabolite pool data set on multiple cultivation conditions, and highlights many interesting observations. It is available at MetaboLights website for further inspection and is a valuable resource for hypothesis generation and design of more targeted studies that can both identify potential new flux regulating metabolites and resolve more about how metabolites both signals and regulates the intracellular state.

## Materials and methods

### Cultivation conditions

*Saccharomyces cerevisiae* CEN.PK 113-7D inoculum was prepared in 6.8 g L^−1^ YNB mineral medium (100 mL) containing 10 g L^−1^ carbon source and grown in baffled flasks at 30 °C with constant shaking at 200 rpm*. S. cerevisiae* was grown in Verduyn medium in batch with four different carbon sources: glucose, fructose, sucrose, and galactose, each having a concentration of 15 g L^−1^, (NH_4_)_2_ SO_4_, 5.0 g L^−1^; KH_2_PO_4_, 3.0 g L^−1^; MgSO4.7H_2_O, 0.5 g L^−1^; 1 mL trace elements and 1 mL vitamin stocks solutions as described in literature [31]. The yeast strain was cultivated in the bioreactor (New Brunswick, BioFlo 115) having a working volume of 1500 mL, maintaining pH 5, 30 °C and air supply of nearly 0.3 vvm and agitation was set to increase in a cascade mode starting from 200 rpm to supply minimum dissolved oxygen level of 40%. The sampling for growth and HPLC analysis (n = 3) was carried out at equal time intervals till the end of the exponential phase of slow-growing carbon source (galactose) and the final sample at the end of fermentation. Sampling for intracellular metabolites (n = 4) was carried out two times: one when optical density (OD) at 600 nm was close to one (exponential phase) and another at a nearly stationary phase.

The chemostat process was run in the bioreactor with a working volume of 1000 mL and had similar operating conditions and medium compositions as described in batch with glucose of 10 g L^−1^ (if specifically, not mentioned) except varying concentration of nutrients (glucose for carbon, ammonium for nitrogen and phosphate for phosphorous limitation) to which chemostat medium was limited. The nitrogen-limited continuous process was started with, 0.4 g L^−1^ (NH_4_)_2_SO_4_ in batch and it was reduced to 0.2 g L^−1^ in the feed tank while the phosphate-limited continuous process was started with 0.1 g L^−1^ KH_2_PO_4_ during batch mode and 0.02 g L^−1^ during chemostat operation. The glucose-limited continuous operation was started with 5 g L^−1^ glucose in batch while the feed tank had 1 g L^−1^ glucose (LG chemostat) during continuous operation. Another glucose-limited chemostat was operated with high glucose, 10 g L^−1^ (HG chemostat) during both batch and chemostat. NaOH (2 M) was used for maintaining pH 5. The sampling (n = 3 for HPLC and n = 4 for intracellular metabolites) in each dilution rate was carried out when the system was in a steady state as measured by OD, stabilization of carbon dioxide profile, and generally taken after four to six bioreactor volume exchange. Two pumps were utilized for automating the feeding of medium and draining of broth while maintaining the same weight of the bioreactor.

### Quantification of extracellular substrates and products

Off gas data (CO_2_, O_2_, gas flow rate) was measured using a Gas Analyzer (DASGIP GA4, BlueSens, Eppendorf). The gas analyzer was calibrated each time before starting an experiment. A known volume of broth was centrifuged at 4500*g* for 5 min at 4 °C. Supernatants were collected for determining concentrations of sugars and organic acids using high-performance liquid chromatography (HPLC) and cell pellets were washed one time with saline water and the second time with MQ water. Biomass was transferred to a pre-weighed aluminum pan and dried at 110 °C until constant weight to prepare a standard curve and to determine dry cell weight (DCW). A standard calibration curve was used to convert OD_600nm_ data into DCW. Extracellular metabolites concentrations were determined using a Hi-Plex column of dimension 300 × 7.7 mm (Agilent Technologies), and a UV/vis and a refractive index (RI) detector on Alliance HPLC **(**Waters**)**. The column was set at 45 °C and metabolites were eluted using 0.05 M H_2_SO_4_ in MQ-H_2_O as mobile phase with a flow rate of 0.8 mL min^−1^. All data are presented as an average of three independent samples.

### Sample preparation for intracellular metabolites quantification

The fast vacuum filtration method was utilized for sampling intracellular metabolites [23]. A known volume of broth (depending upon cell density) was vacuum filtered using Supor^®^ hydrophilic polyethersulfone filter having a pore size of 0.8 µm, 47 mm at 400 mbar below ambient pressure and washed quickly with precooled saline water (0.9%, w/v), followed by milliQ water. The filter containing cell biomass was immediately quenched in a known volume of pre-cooled extraction solvent in a 50 mL centrifuge tube having the composition of Acetonitrile 55% and Water 45%. The quenched samples were placed in − 80 °C until processed. All samples were processed for extracting intracellular metabolites into extracting solvent by three times freeze-thawing cycle: freezing in liquid nitrogen followed by thawing at nearly 0 °C in ethanol bath. A brief vortexing of tubes was introduced between each freeze-thawing cycle. The extraction solvent was centrifuged for 5 min at 4500*g* and 4 °C to separate filter/cell debris and supernatant. The extraction solvent containing intracellular metabolites was distributed into three aliquots into 15 mL Eppendorf tubes and concentrated by lyophilization and stored at − 80 °C. Each aliquot containing intracellular metabolites dried powder was reconstituted in 550 µL cold HPLC grade water, spin filtered in 3 kDa molecular cutoff filter using a centrifuge at 4 °C, and 20,000* g*.

### Intracellular metabolites quantification

Intracellular metabolite pools were analyzed on an ACQUITY UPLC system coupled to a Xevo TQXS triple quadrupole mass spectrometer equipped with an electrospray source (Waters, Milford, MA, USA) and each aliquoted sample was quantified using three tandem mass spectrometry (MS/MS) methods: two UPLC-MS/MS methods each for amino acids and organic acids, a capillary ion chromatography (capIC-MS/MS) method for nucleotides, sugar phosphates and other phosphometabolites [17, 23, 45]. An electrospray source was set in the negative mode for capIC-MS/MS method. The isotope dilution strategy was used for nullifying matrix effect and external standards/samples were diluted by 20% using an internal standard. The biological extract of *S cerevisiae* grown on U^13^C-labeled glucose was used as internal standards in both organic acid and CapIC methods.

The organic acid method was adapted from literature and O-benzylhydroxylamine (O-BHA) and N-(3-Dimethylaminopropyl)-N′-ethylcarbodiimide hydrochloride (EDAC) were used as derivatizing reagents. The external standards and samples were derivatized in a 96 well plate format. An electrospray source was set in positive mode. The organic acid metabolites were separated using Waters Acquity BEH C18 column having the dimension of 2.1 × 100 mm and pore size of 1.7 µm (Waters) maintained at 40 °C and eluted with a gradient of mobile phases: HPLC-grade water with 0.1% formic acid and pure methanol.

As in organic acids, external standards and samples were derivatized in a 96 well plate format using phenyl isothiocyanate as derivatization reagent (PITC methods). The ^13^C, ^15^ N isotope-labeled (Cambridge Isotope Laboratories) amino acids mixture containing 19 amino acids was used as an internal standard in amino acids estimation. Derivatized samples were separated using an ACQUITY UPLC BEH C18 column having the dimension of 2.1 × 75 mm fitted with an ACQUITY UPLC BEH C18 2.1 × 5 mm VanGuard pre-column, both with a pore size of 1.7 µm (Waters). The column was set at 50 °C and eluted with a gradient of mobile phases: HPLC-grade water and acetonitrile, both containing 0.2% formic acid**.**

### Computation and statistical analysis

Specific growth rate, substrate consumption rate, and extracellular metabolites production rates were calculated in the exponential phase of the batch by a linear least square regression between lnX and (t–t_0_) for specific growth rate and between (S or P) and (X–X_0_) for consumption and production rates, where X, S, P are DCW, substrate, and extracellular metabolite concentration at the time, t respectively and t_0_ and X_0_ are initial time and biomass, respectively. Mass spectrometry data were acquired using MassLynx 4.2 (Waters) and data was processed and quantified using its application manager, TargetLynx 4.2. All intracellular metabolites pools were reported as an average of three to four independent replicas. Heat-maps and principal component analysis (PCA) score plots were generated using the MetaboAnalyst (Xia and Wishart 2016) and the R statistical software. For ease of analysis, missing data were substituted with zeroes. The correlation/principal component analysis was carried out using the factoextra package in R. The correlation analysis between substrate consumption rate and metabolite pools was performed with Excel. First was all data sorted on increasing subsrate consumption rate, then slope and r square values determined, and finally sorted on decreasing r square values as presented in Additional file 6: Table S9. Prior to performing the PCA, the data was autoscaled to zero mean and unit standard deviation. The statistical analysis was conducted using a two-tailed t-test (assuming unequal variance) on the metabolome data set of the selected cultivation condition to the reference data set. The central metabolism is visualized using the Omix software [46].

## Supplementary Information

**Additional file 1: Table S1.** Growth performance of *S. cerevisiae* in exponential phase of batch when cultivated in different carbon sources. **Table S2.** Growth parameters of *S. cerevisiae* in carbon (glucose) limited chemostat (Feed glucose concentration (HG) = 10 g L^-1^). **Table S3.** Growth parameters of *S. cerevisiae* in carbon (glucose) limited chemostat (Feed glucose concentration (LG) = 1 g L^-1^) **Table S4.** Growth parameters of *S. cerevisiae* in nitrogen (ammonium) limited chemostat. **Table S5.** Growth parameters of *S. cerevisiae* in phosphorous (phosphate) limited chemostat

**Additional file 2: Figure S1.** CO_2_ evolution and O_2_ consumption curve of *Saccharomyces cerevisiae* CENPK when cultivated in batch using different carbon sources **A)** Glucose **B)** Fructose **C)** Sucrose **D)** Galactose . **Figure S2.** Heat-map showing relative standard deviation of intracellular metabolites for four independent samples. **Figure S3.** Metabolites variations (symbol, left and right error indicate average, minimum and maximum value of metabolite, respectively) across different cultivation conditions under current study on absolute concentration scale (Left panel), and log2 of lowest (left bar range) and highest (right bar range) concentration, normalized by subtracting log2 of average for each metabolite from their lowest and highest value (Right panel).** Figure S4. **PCA scores (upper panel) and loadings (lower panel, top 50 metabolites) plots of merged extracellular rates - and endometabolite data from all cultivations except stationary batch phase. Gal1P and Gal6P were removed from analysis since <LOD for most conditions and data was normalized to sum and autoscaled before analysis. **Figure S5.** Spearman rank correlation of combined exo and endo metabolite data for all cultivation conditions, except batch stationary phase. **Figure S6.** Spearman rank correlation of combined exo and endo metabolite data for the four batch growth phase conditions. **Figure S7.** Spearman rank correlation of combined exo and endo metabolite data for the Low Glucose limited chemostats. **Figure S8.** Spearman rank correlation of combined exo and endo metabolite data for the High Glucose limited chemostats. **Figure S9.** Spearman rank correlation of combined exo and endo metabolite data for the Nitrogen limited chemostats.** Figure S10.** Spearman rank correlation of combined exo and endo metabolite data for the Phosphate limited chemostats. **Figure S11.** A plot showing changes in the metabolites of central metabolic pathways. This plot is plotted based on logarithmic value (base 2) of ratio of intracellular metabolites concentration of yeast cultivated on galactose to yeast cultivated on glucose’ in exponential phase of batch operation. GAL1P is highly expressed in galactose but not detected in glucose grown yeast in batch reasoning gray color in plot. **Figure S12.** Substrate consumption vs. growth rate for all cultivation conditions. Glim is divided into two categories, those with no ethanol production and the two at highest dilution, i.e growth rate, where ethanol production were detected.

**Additional file 3.**

**Additional file 4.**

**Additional file 5: **Table S8. t-test of pairwise comparisons (paired, 2-tailed) in Panel A-G in Figure 2. Yellow asterix indicate p < 0.05.

**Additional file 6: **Table S9. Correlation between carbon source consumption rate and and other rates and metabolite pools. Color formatting of rates and metabolites mentioned in the main text.

## Data Availability

All processed metabolite data is available in the main text or the additional material, and raw data files can be accessed by contacting corresponding author. Metabolite abbreviations can be found in Additional file 6: Table S6.
