# Supplementary material for: Adaptation of central metabolite pools to variations in growth rate and cultivation conditions in Saccharomyces cerevisiae
Source: Microb Cell Fact. 2021 Mar 9;20:64. doi: 10.1186/s12934-021-01557-8 (PMC7941957; doi:10.1186/s12934-021-01557-8)
Supplement: Supplementary file 1 — Additional file 1: Table S1. Growth performance of S. cerevisiae in exponential phase of batch when cultivated in different carbon sources. Table S2. Growth parameters of S. cerevisiae in carbon (glucose) limited chemostat (Feed glucose concentration (HG) = 10 g L-1). Table S3. Growth parameters of S. cerevisiae in carbon (glucose) limited chemostat (Feed glucose concentration (LG) = 1 g L-1) Table S4. Growth parameters of S. cerevisiae in nitrogen (ammonium) limited chemostat. Table S5. Growth parameters of S. cerevisiae in phosphorous (phosphate) limited chemostat [file 12934_2021_1557_MOESM1_ESM.docx]

**Additional file 1: Table S1.** Growth performance of *S. cerevisiae* in exponential phase of batch when cultivated in different carbon sources.

| Carbon source | µ | Y_xs_ | q_s_ | Y_EtOHs_ | q_EtOH_ | q_CO2_ | q_O2_ | C^#^ recovery (%) |
| --- | --- | --- | --- | --- | --- | --- | --- | --- |
|  | (h^-1^) | (g DCW | (g g^-1^ DCW h^-1^) | (g EtOH | (g g^-1^ DCW h^-1^) | (mmol g^-1^ DCW h^-1^) | (mmol g^-1^ DCW h^-1^) |  |
|  |  | g^-1^ glucose) |  | g^-1^ glucose) |  |  |  |  |
| Glucose | 0.43± 0.004 | 0.13±  0.01 | 3.35±  0.18 | 0.49±  0.08 | 1.65±  0.27 | 29.50± 1.00 | 13.76± 0.47 | 95.0±  7.45 |
|  |  |  |  |  |  |  |  |  |
| Fructose | 0.42± 0.004 | 0.14±  0.01 | 4.23±  0.21 | 0.28±  0.03 | 1.2±  0.11 | 30.53± 1.05 | 17.05± 0.59 | 104.0± 5.27 |
|  |  |  |  |  |  |  |  |  |
| Sucrose^a^ | 0.41± 0.004 | 0.13±  0.01 | 3.2±  0.13 | 0.54±  0.10 | 1.72±  0.30 | 34.73± 1.38 | 14.97± 0.60 | 103.0± 9.14 |
|  |  |  |  |  |  |  |  |  |
| Galactose | 0.26± 0.004 | 0.26±  0.01 | 0.96±  0.03 | 0.29±  0.01 | 0.28±  0.01 | 11.89± 0.30 | 8.94±  0.22 | 102.0± 1.20 |
|  |  |  |  |  |  |  |  |  |

^a^ = Calculated in terms of glucose

^#^ = carbon recovery at the end of exponential phase

**Additional file 1: Table S2.** Growth parameters of *S. cerevisiae* in carbon (glucose) limited chemostat (Feed glucose concentration (HG) = 10 g L^-1^)

| D | DCW (g L^-1^) | Y_xs_ | q_s_ | Y_EtOHS_ | q_EtOH_ | q_CO2_ | q_O2_ | RQ | C recovery (%) |
| --- | --- | --- | --- | --- | --- | --- | --- | --- | --- |
| (h^-1^) |  | (g DCW g^-1^ glucose) | (g g^-1^ DCW h^-1^) | (g ETOH g^-1^ glucose) | (g g^-1^ DCW h^-1^) | (mmol g^-1^ DCW h^-1^) | (mmol g^-1^ DCW h^-1^) |  |  |
|  |  |  |  |  |  |  |  |  |  |
| 0.12 | 4.25± 0.09 | 0.43± 0.01 | 0.28± 0.01 | 0.0± 0.00 | 0.0± 0.00 | 3.50± 0.10 | 3.35± 0.00 | 1.0± 0.03 | 92.2± 1.93 |
| 0.26 | 4.40± 0.24 | 0.44± 0.03 | 0.59± 0.03 | 0.0± 0.00 | 0.0± 0.00 | 5.84± 0.33 | 5.60± 0.31 | 1.0± 0.01 | 88.0± 4.68 |
| 0.35 | 1.71± 0.05 | 0.17± 0.01 | 2.05± 0.05 | 0.28± 0.00 | 0.57± 0.00 | 19.26± 0.66 | 10.14± 0.11 | 1.9± 0.07 | 86.6± 1.01 |
| 0.41 | 0.55± 0.00 | 0.11± 0.00 | 3.76± 0.06 | 0.35± 0.04 | 1.31± 0.15 | 26.68± 0.20 | 9.37± 0.04 | 2.9± 0.06 | 82.2± 6.55 |

**Additional file 1: Table S3.** Growth parameters of *S. cerevisiae* in carbon (glucose) limited chemostat (Feed glucose concentration (LG) = 1 g L^-1^)

| D | DCW | Y_xs_ | q_s_ | Y_EtOHS_ | q_EtOH_ | q_CO2_ | q_O2_ | RQ | C recovery (%) |
| --- | --- | --- | --- | --- | --- | --- | --- | --- | --- |
| (h^-1^) | (g L^-1^) | (g DCW g^-1^ glucose) | (g g^-1^ DCW h^-1^) | (g ETOH g^-1^ glucose) | (g g^-1^ DCW h^-1^) | (mmol g^-1^ DCW h^-1^) | (mmol g^-1^ DCW h^-1^) |  |  |
|  |  |  |  |  |  |  |  |  |  |
| 0.05 | 0.32± 0.02 | 0.32± 0.02 | 0.16± 0.01 | 0.00± 0.00 | 0.00± 0.00 | 2.28± 0.12 | 2.42± 0.13 | 0.9± 0.01 | 83.1± 4.28 |
| 0.12 | 0.44± 0.03 | 0.44± 0.03 | 0.27± 0.02 | 0.00± 0.02 | 0.00± 0.01 | 3.95±  0.26 | 3.47± 0.23 | 1.1± 0.03 | 107.1± 6.23 |
| 0.18 | 0.34± 0.01 | 0.34± 0.01 | 0.53± 0.02 | 0.00± 0.04 | 0.00± 0.03 | 7.52±  0.25 | 7.51± 0.25 | 1.0± 0.01 | 86.1± 2.33 |
| 0.24 | 0.33± 0.02 | 0.33± 0.02 | 0.73± 0.04 | 0.00± 0.02 | 0.00± 0.03 | 8.30±  0.51 | 8.11± 0.50 | 1.0± 0.02 | 76.7± 3.85 |
| 0.31 | 0.30± 0.01 | 0.30± 0.01 | 1.03± 0.03 | 0.00± 0.00 | 0.00± 0.04 | 10.10±  0.30 | 10.45± 0.31 | 1.0± 0.02 | 67.6± 1.44 |
|  |  |  |  |  |  |  |  |  |  |

**Additional file 1: Table S4.** Growth parameters of *S. cerevisiae* in nitrogen (ammonium) limited chemostat

| D | DCW (g L^-1^) | Y_xs_ | q_s_ | Y_EtOHS_ | q_EtOH_ | q_CO2_ | q_O2_ | RQ | C recovery (%) |
| --- | --- | --- | --- | --- | --- | --- | --- | --- | --- |
| (h^-1^) |  | (g DCW g^-1^ glucose) | (g g^-1^ DCW h^-1^) | (g ETOH g^-1^ glucose) | (g g^-1^ DCW h^-1^) | (mmol g^-1^ DCW h^-1^) | (mmol g^-1^ DCW h^-1^) |  |  |
|  |  |  |  |  |  |  |  |  |  |
|  |  |  |  | 0.31± 0.00 |  |  |  |  |  |
| 0.06 | 1.34± 0.09 | 0.16± 0.01 | 0.37± 0.03 |  | 0.12± 0.00 | 4.77± 0.34 | 3.23± 0.23 | 1.5±  0.03 | 105.3± 4.11 |
|  |  |  |  |  |  |  |  |  |  |
|  |  |  |  | 0.33± 0.02 |  |  |  |  |  |
| 0.12 | 1.20± 0.03 | 0.17± 0.01 | 0.69± 0.05 |  | 0.23± 0.01 | 8.42± 0.21 | 4.14± 0.10 | 2.0±  0.02 | 104.3± 10.5 |
|  |  |  |  |  |  |  |  |  |  |
|  |  |  |  | 0.31± 0.04 |  |  |  |  |  |
| 0.19 | 0.81± 0.03 | 0.15± 0.01 | 1.26± 0.08 |  | 0.39± 0.03 | 15.32± 0.63 | 5.84± 0.24 | 2.6±  0.06 | 100.6± 6.40 |
|  |  |  |  |  |  |  |  |  |  |
|  |  |  |  | 0.38± 0.02 |  |  |  |  |  |
| 0.24 | 0.62± 0.03 | 0.16± 0.01 | 1.47± 0.07 |  | 0.56± 0.03 | 18.06± 0.85 | 6.76± 0.32 | 2.7±  0.05 | 111.5± 4.25 |
|  |  |  |  |  |  |  |  |  |  |
|  |  |  |  | 0.46± 0.00 |  |  |  |  |  |
| 0.32 | 0.52± 0.02 | 0.15± 0.01 | 2.21± 0.09 |  | 1.03± 0.04 | 21.97± 0.86 | 8.04± 0.32 | 2.7±  0.05 | 112.6± 11.71 |
|  |  |  |  |  |  |  |  |  |  |
|  |  |  |  | 0.44± 0.00 |  |  |  |  |  |
| 0.34 | 0.46± 0.02 | 0.11± 0.01 | 3.12± 0.27 |  | 1.39± 0.04 | 27.63± 1.14 | 9.52± 0.39 | 2.9±  0.06 | 101.0± 11.11 |
|  |  |  |  |  |  |  |  |  |  |

**Additional file 1: Table S5.** Growth parameters of *S. cerevisiae* in phosphorous (phosphate) limited chemostat

| D | DCW (g L^-1^) | Y_xs_ | q_s_ | Y_EtOHS_ | q_EtOH_ | q_CO2_ | q_O2_ | RQ | C recovery (%) |
| --- | --- | --- | --- | --- | --- | --- | --- | --- | --- |
| (h^-1^) |  | (g DCW g^-1^ glucose) | (g g^-1^ DCW h^-1^) | (g ETOH g^-1^ glucose) | (g g^-1^ DCW h^-1^) | (mmol g^-1^ DCW h^-1^) | (mmol g^-1^ DCW h^-1^) |  |  |
|  |  |  |  |  |  |  |  |  |  |
| 0.06 | 0.37± 0.02 | 0.05± 0.00 | 1.13± 0.08 | 0.24± 0.01 | 0.28± 0.01 | 15.37± 0.82 | 10.97± 0.58 | 1.4±  0.02 | 83.4± 2.32 |
| 0.12 | 0.55± 0.02 | 0.08± 0.01 | 1.52± 0.14 | 0.43± 0.04 | 0.65± 0.03 | 20.76± 0.61 | 10.61± 0.31 | 2.0±  0.02 | 98.2± 9.20 |
| 0.18 | 0.32± 0.04 | 0.06± 0.01 | 3.01± 0.36 | 0.47± 0.02 | 1.41± 0.03 | 20.34± 2.42 | 7.55± 0.90 | 2.7±  0.08 | 92.8± 2.52 |
