# Supplementary material for: Adaptation of central metabolite pools to variations in growth rate and cultivation conditions in Saccharomyces cerevisiae
Source: Microb Cell Fact. 2021 Mar 9;20:64. doi: 10.1186/s12934-021-01557-8 (PMC7941957; doi:10.1186/s12934-021-01557-8)
Supplement: Supplementary file 2 — Additional file 2: Figure S1. CO2 evolution and O2 consumption curve of Saccharomyces cerevisiae CENPK when cultivated in batch using different carbon sources A) Glucose B) Fructose C) Sucrose D) Galactose . Figure S2. Heat-map showing relative standard deviation of intracellular metabolites for four independent samples. Figure S3. Metabolites variations (symbol, left and right error indicate average, minimum and maximum value of metabolite, respectively) across different cultivation conditions under current study on absolute concentration scale (Left panel), and log2 of lowest (left bar range) and highest (right bar range) concentration, normalized by subtracting log2 of average for each metabolite from their lowest and highest value (Right panel). Figure S4. PCA scores (upper panel) and loadings (lower panel, top 50 metabolites) plots of merged extracellular rates - and endometabolite data from all cultivations except stationary batch phase. Gal1P and Gal6P were removed from analysis since <LOD for most conditions and data was normalized to sum and autoscaled before analysis. Figure S5. Spearman rank correlation of combined exo and endo metabolite data for all cultivation conditions, except batch stationary phase. Figure S6. Spearman rank correlation of combined exo and endo metabolite data for the four batch growth phase conditions. Figure S7. Spearman rank correlation of combined exo and endo metabolite data for the Low Glucose limited chemostats. Figure S8. Spearman rank correlation of combined exo and endo metabolite data for the High Glucose limited chemostats. Figure S9. Spearman rank correlation of combined exo and endo metabolite data for the Nitrogen limited chemostats. Figure S10. Spearman rank correlation of combined exo and endo metabolite data for the Phosphate limited chemostats. Figure S11. A plot showing changes in the metabolites of central metabolic pathways. This plot is plotted based on logarithmic value (base 2) of ratio of intracellular metab [file 12934_2021_1557_MOESM2_ESM.docx]

**Additional file 2: Figures Kumar et al “Adaptation of central metabolite pools to variations in growth rate and cultivation conditions in *Saccharomyces cerevisiae*”**


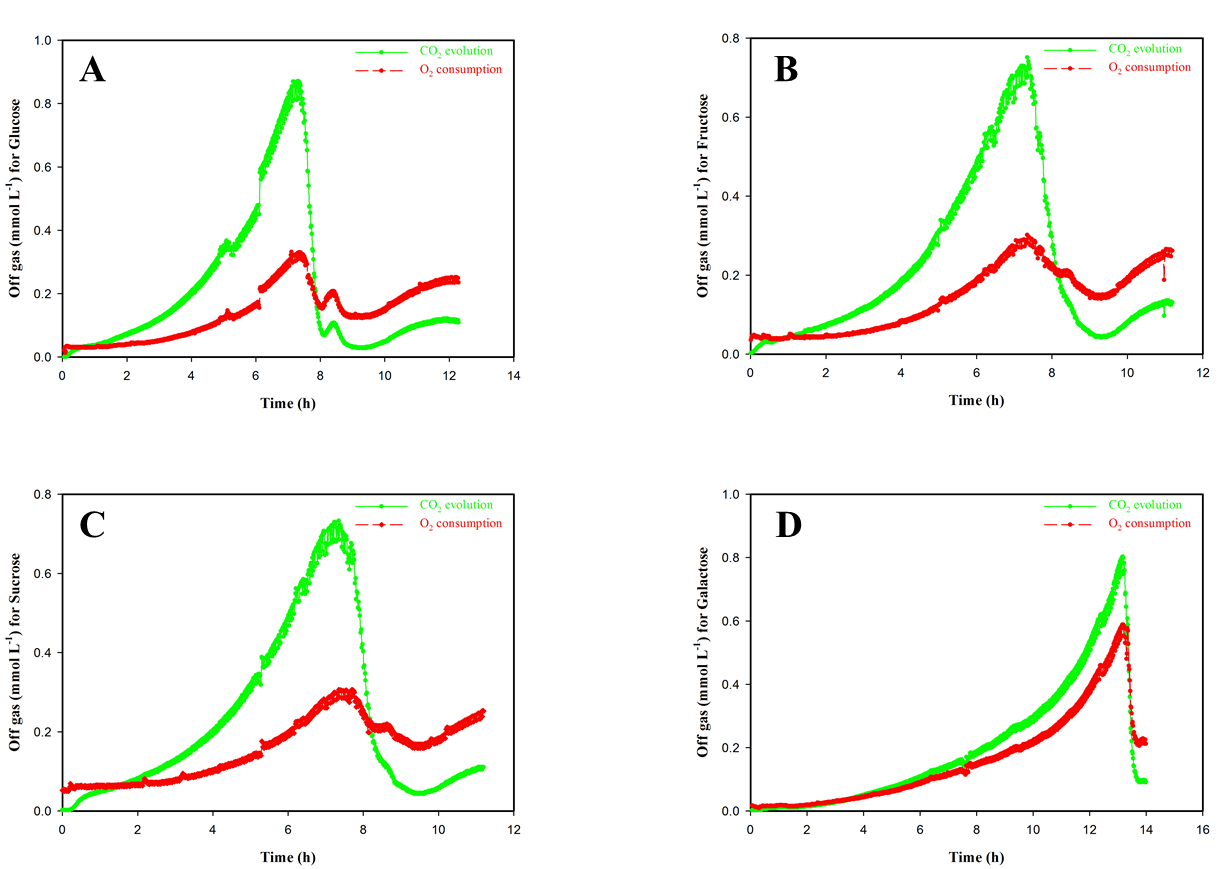


**Additional file 2: Figure S1.** CO_2_ evolution and O_2_ consumption curve of *Saccharomyces cerevisiae* CENPK when cultivated in batch using different carbon sources **A)** Glucose **B)** Fructose **C)** Sucrose **D)** Galactose


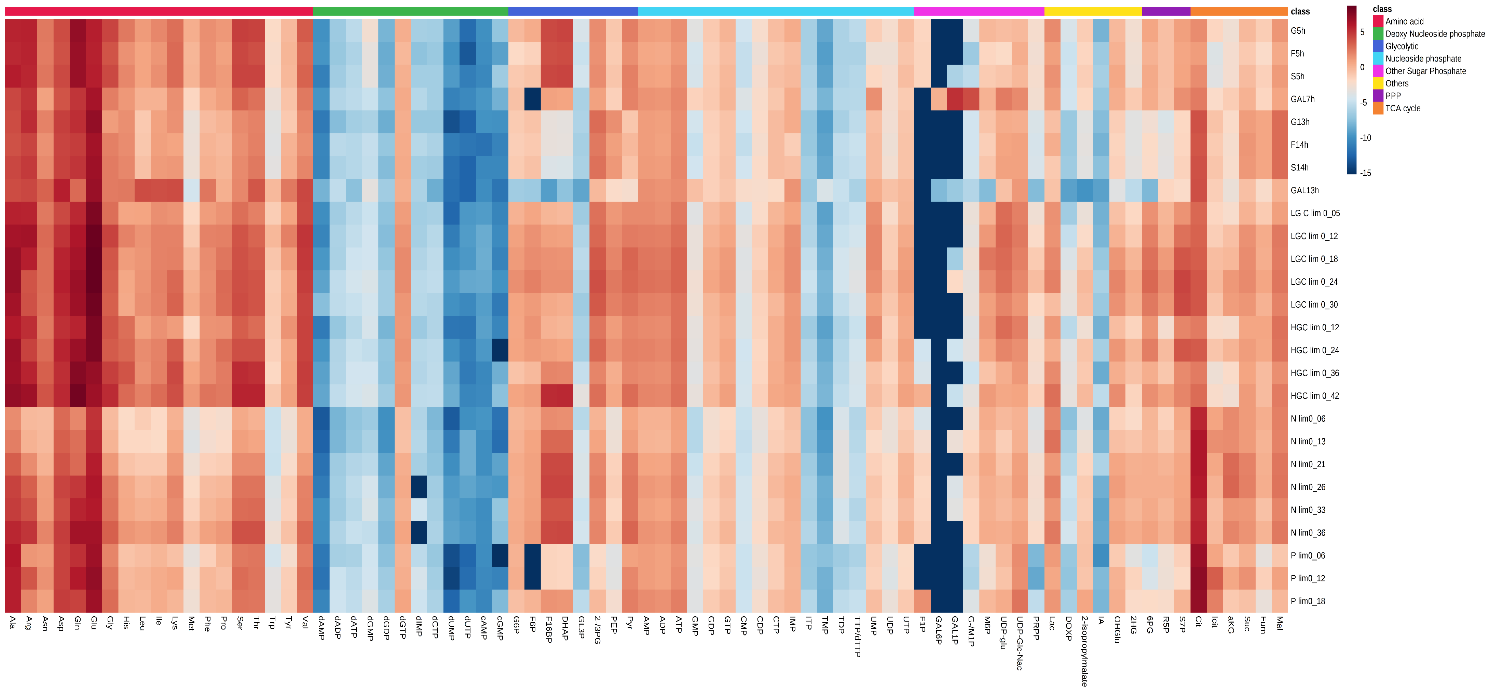

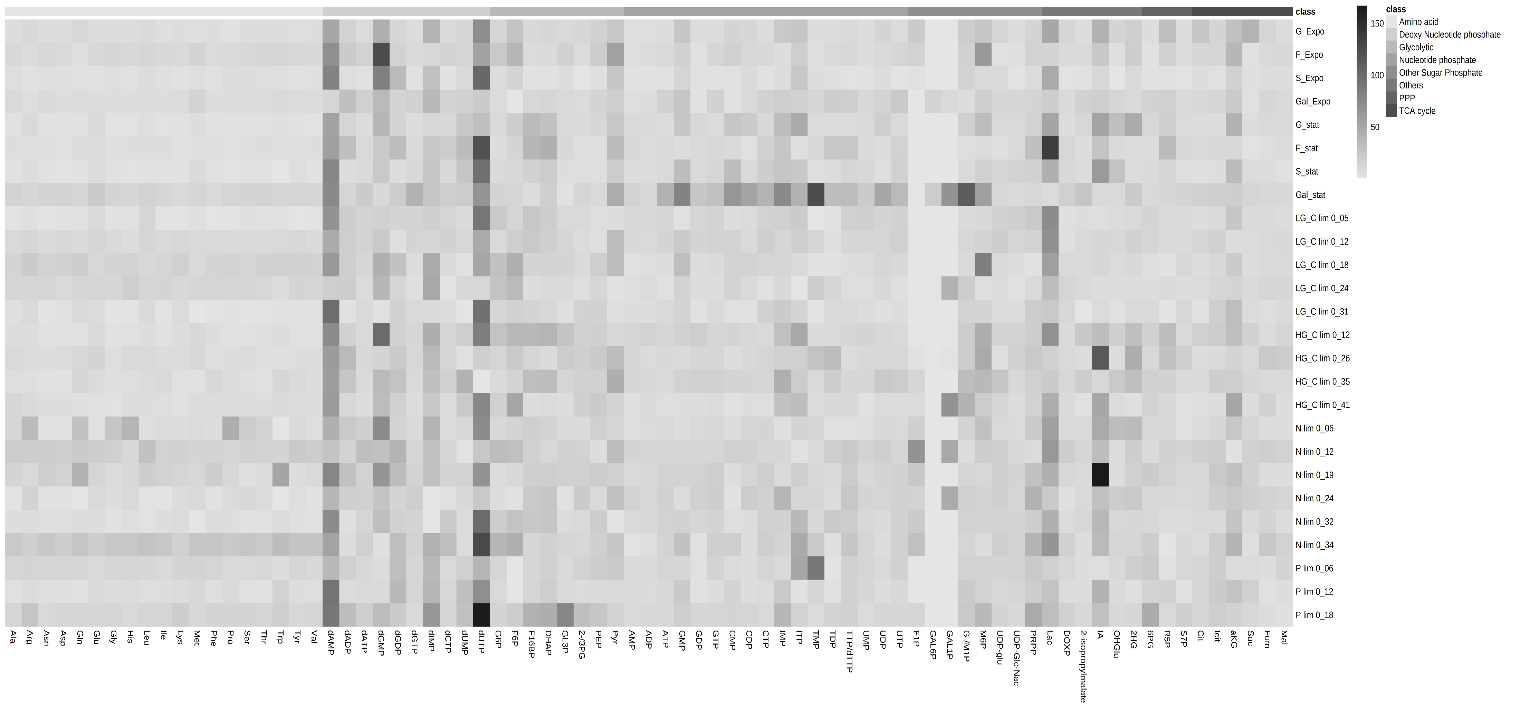

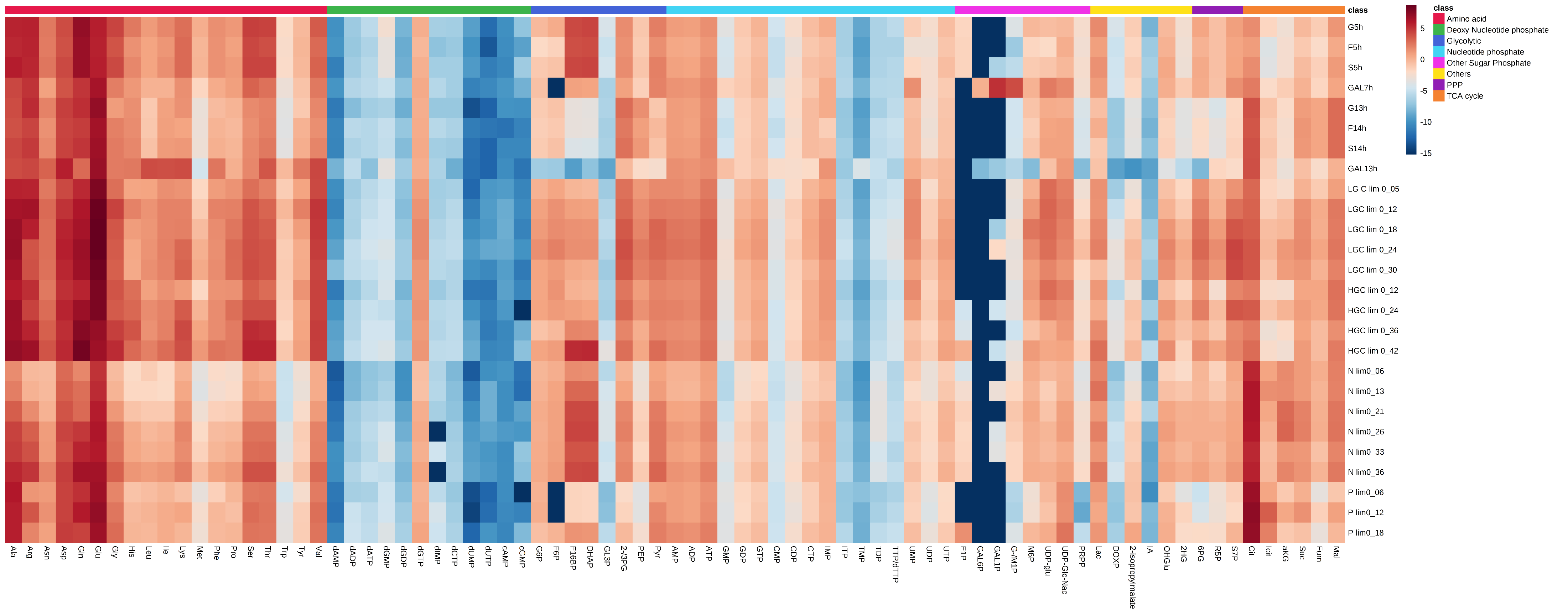

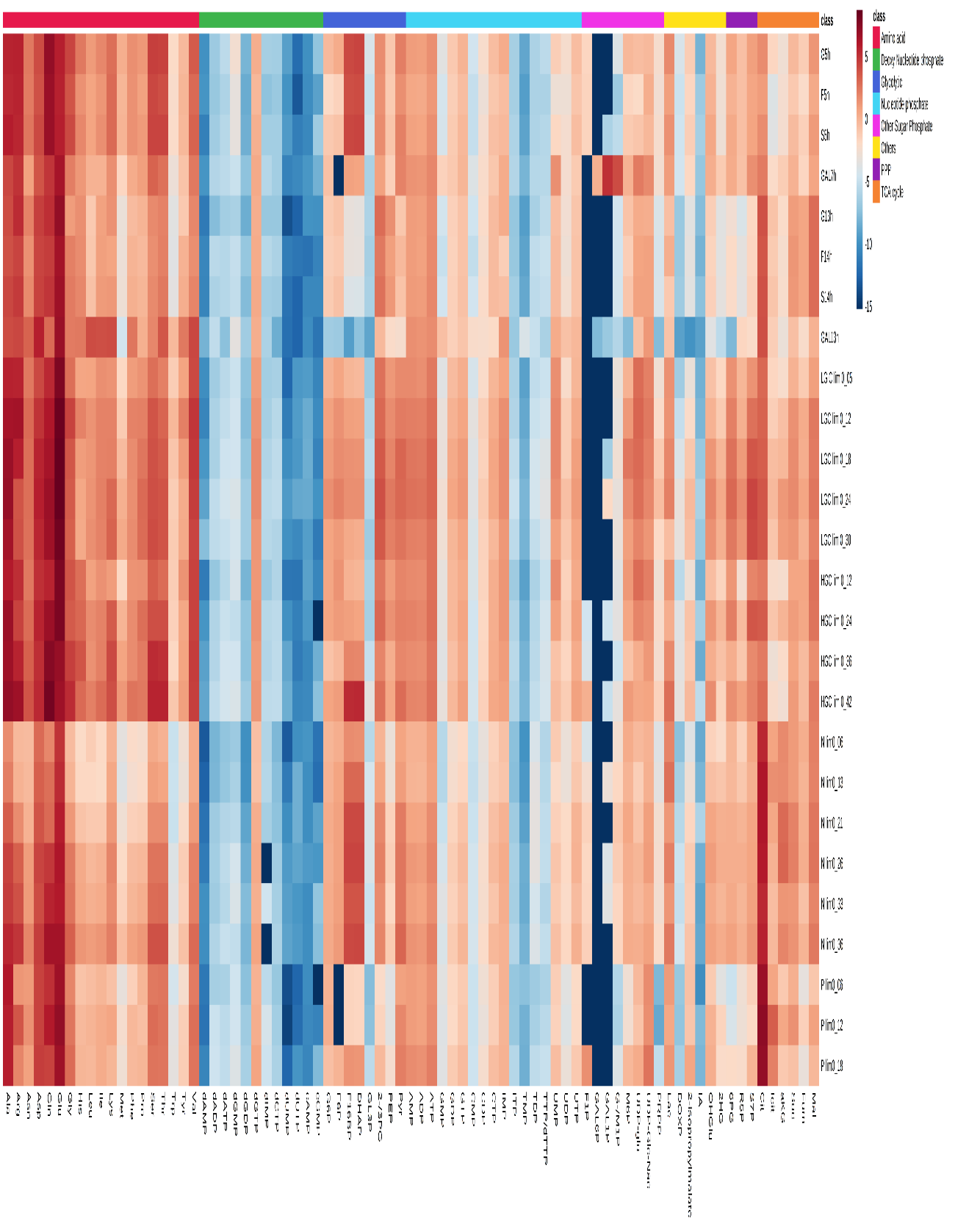

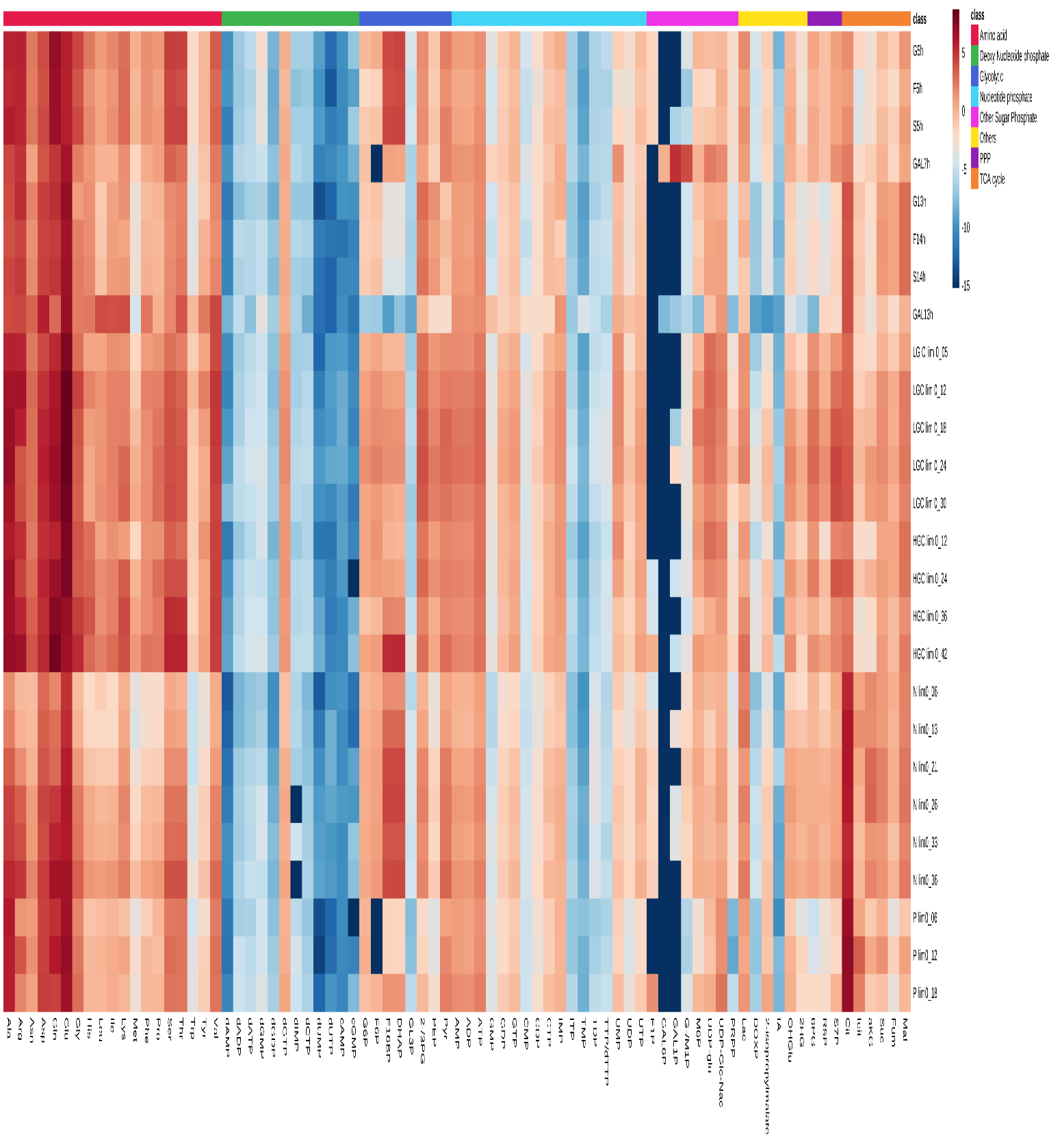

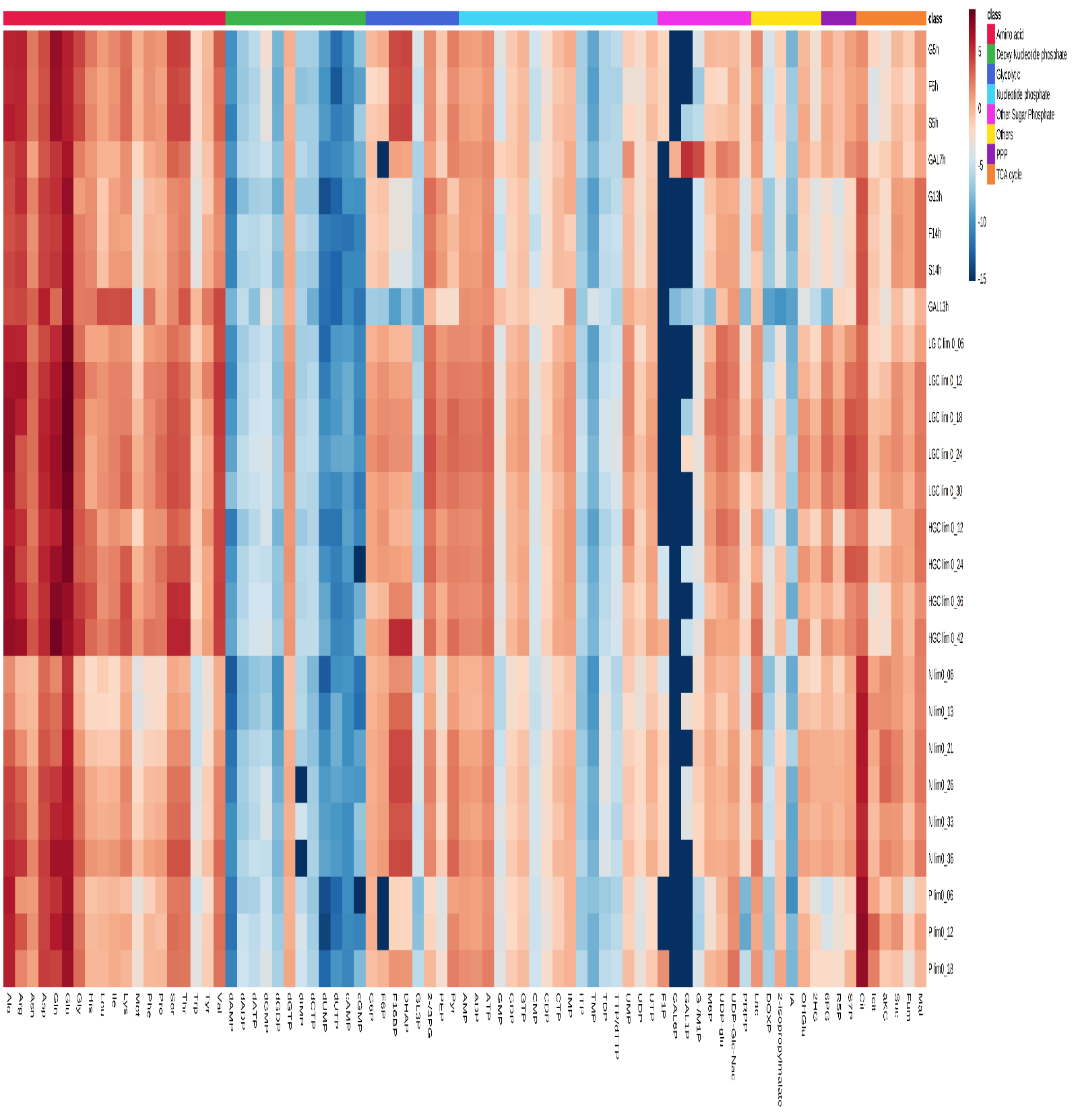

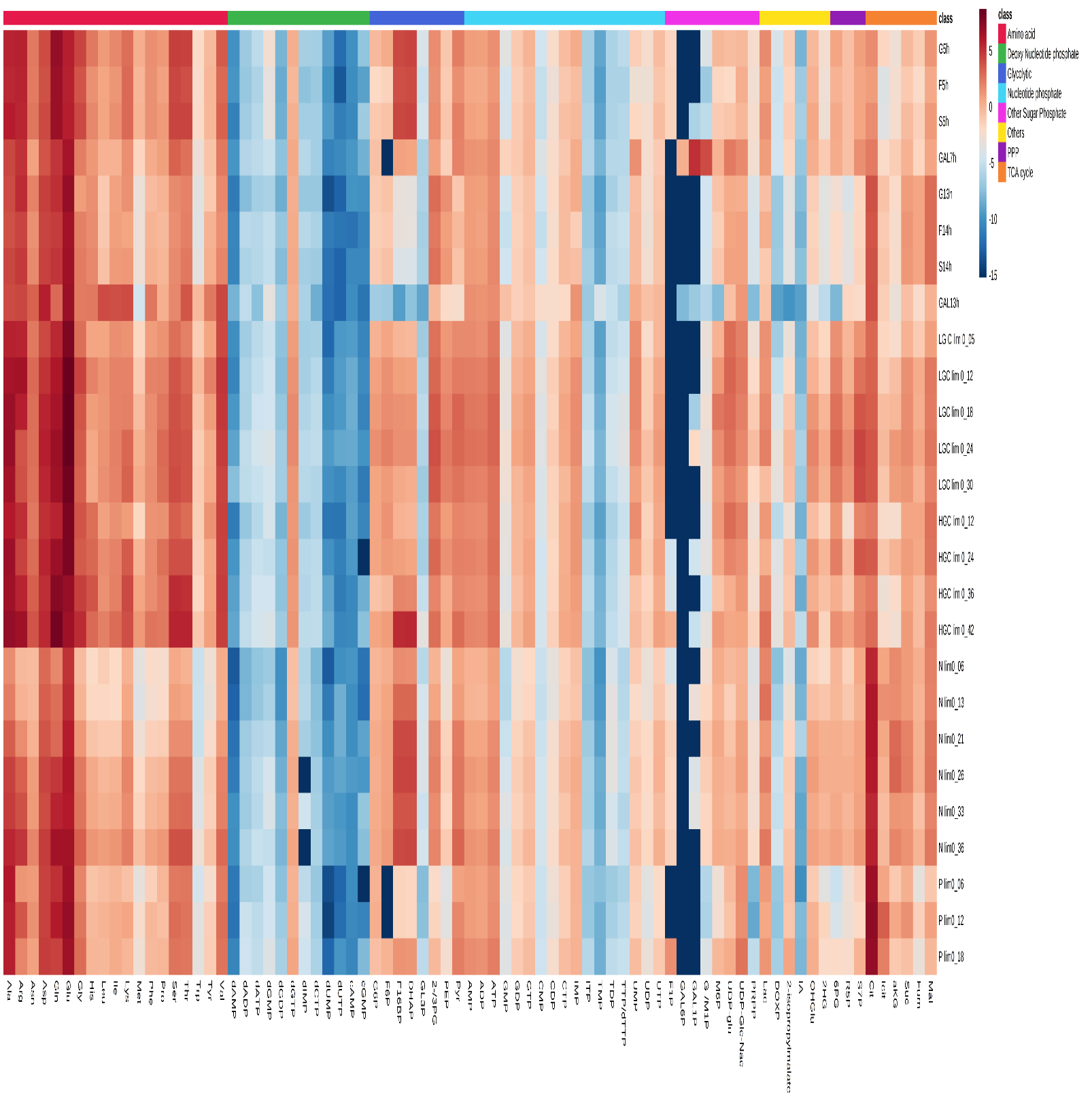

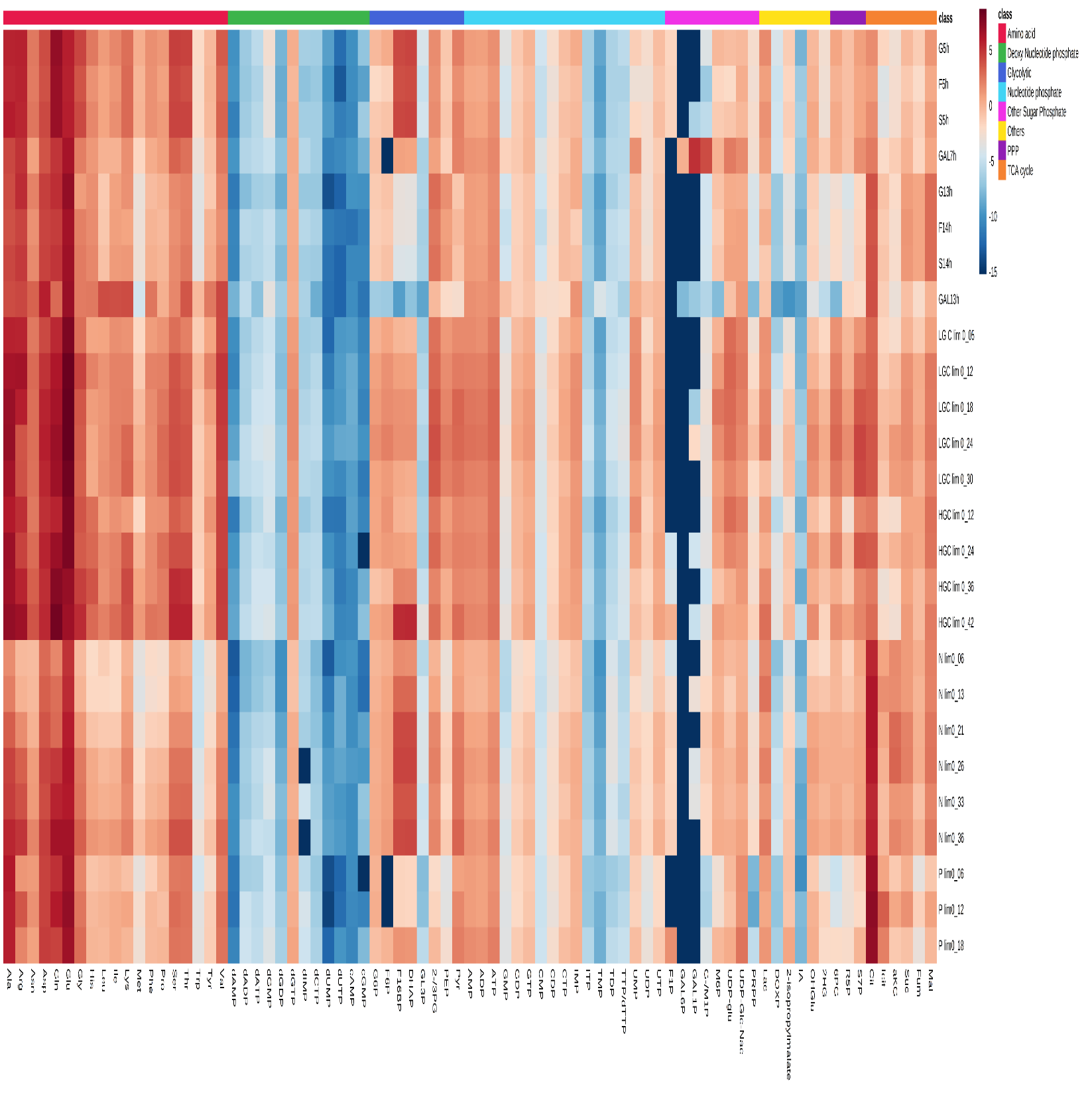

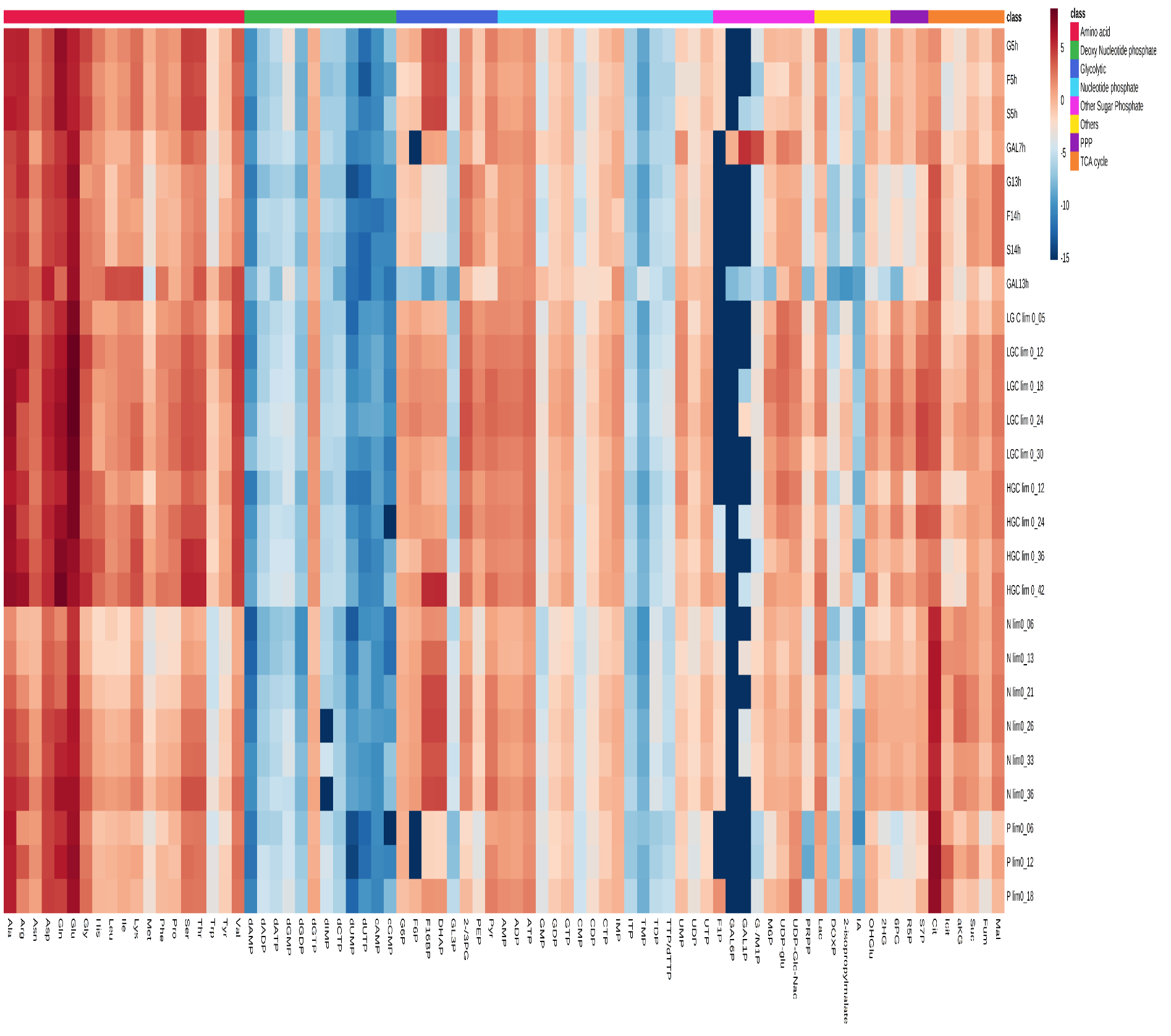

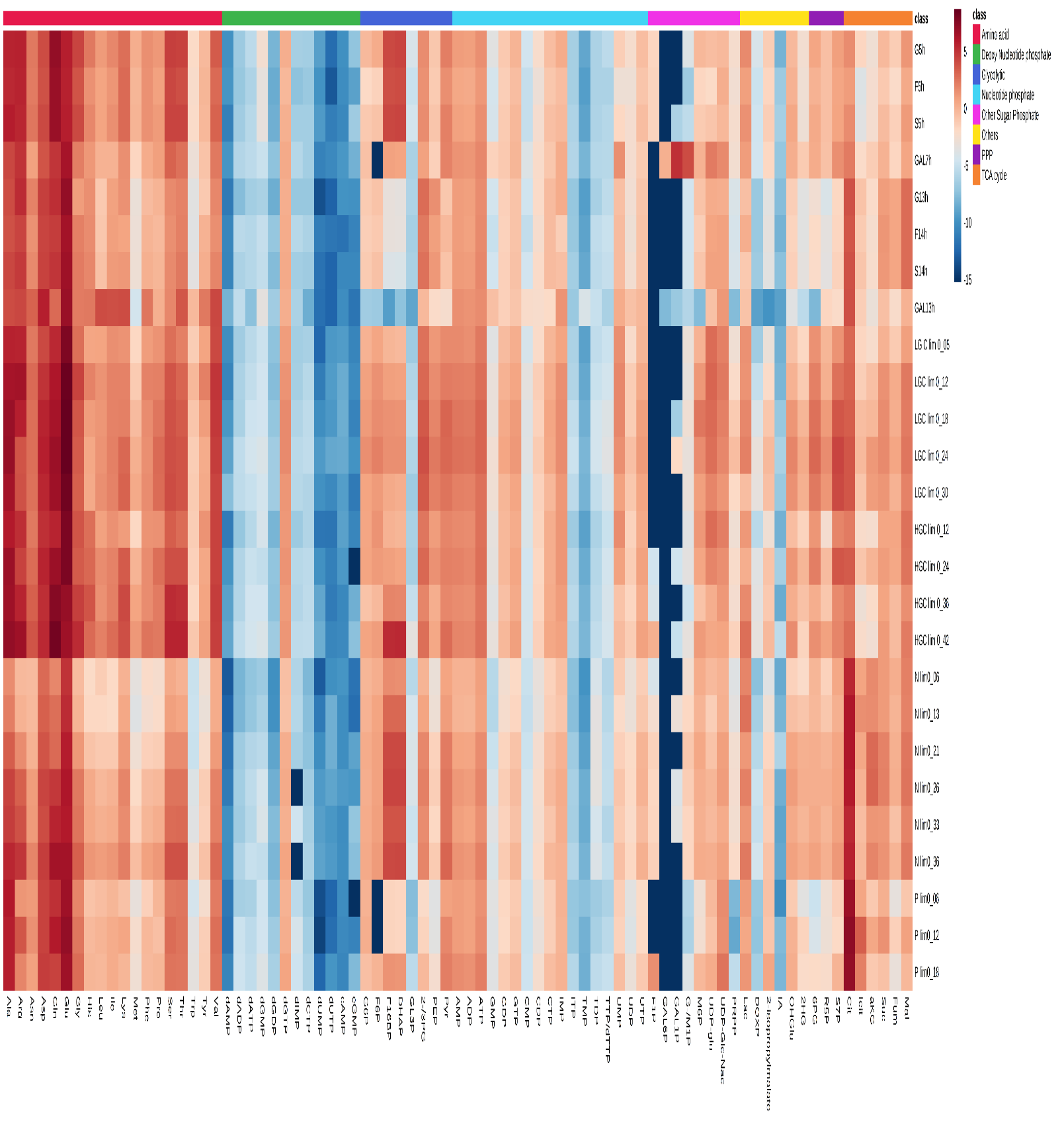


**Additional file 2: Figure S2.** Heat-map showing relative standard deviation of intracellular metabolites for four independent samples.

**Suppmentary Figure S3.** Metabolites variations (symbol, left and right error indicate average, minimum and maximum value of metabolite, respectively) across different cultivation conditions under current study on absolute concentration scale (Left panel), and log2 of lowest (left bar range) and highest (right bar range) concentration, normalized by subtracting log2 of average for each metabolite from their lowest and highest value (Right panel).

**
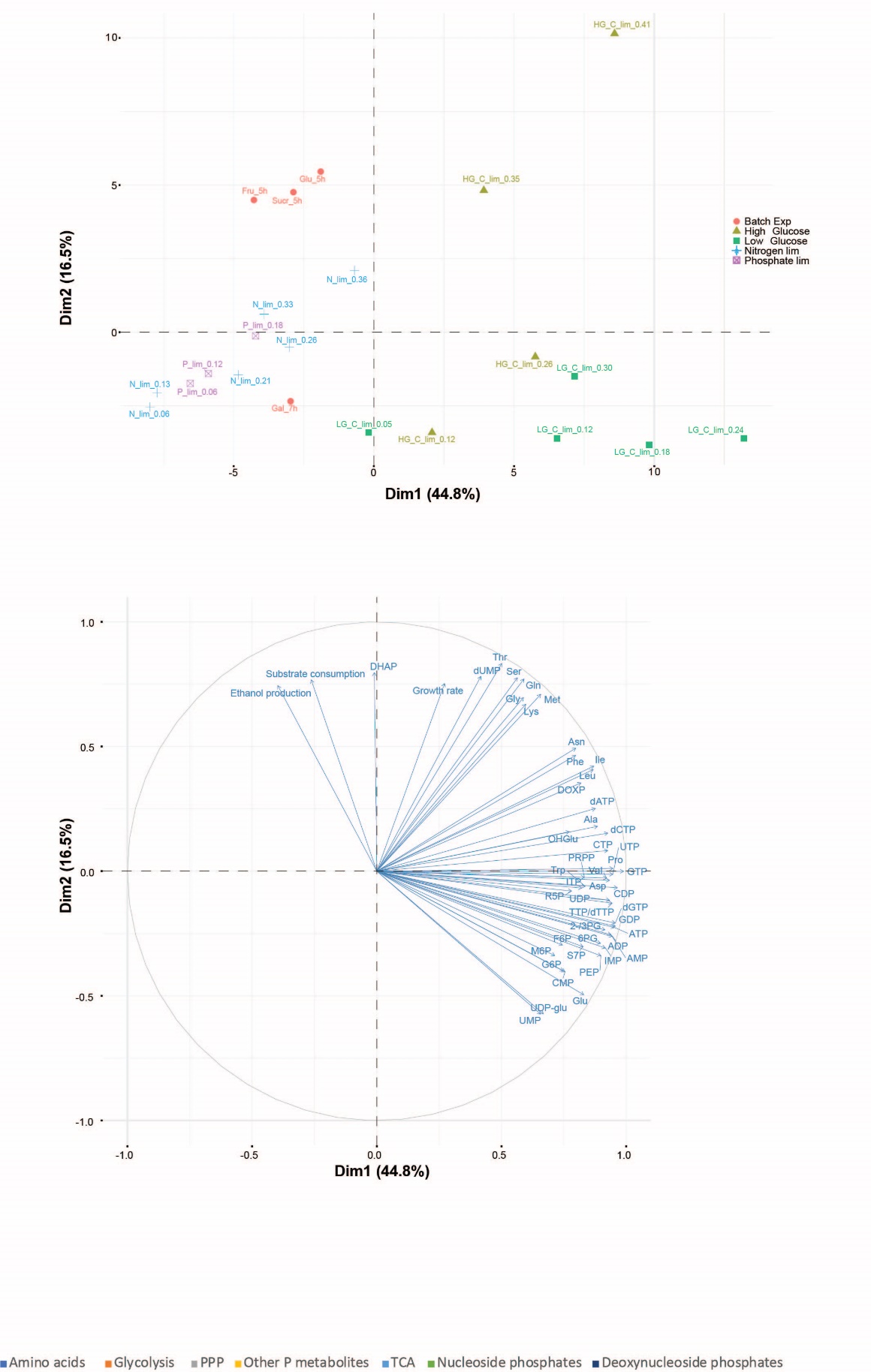
 Additional file 2: Figure S4.** PCA scores (upper panel) and loadings (lower panel, top 50 metabolites) plots of merged extracellular rates - and endometabolite data from all cultivations except stationary batch phase. Gal1P and Gal6P were removed from analysis since <LOD for most conditions and data was normalized to sum and autoscaled before analysis.


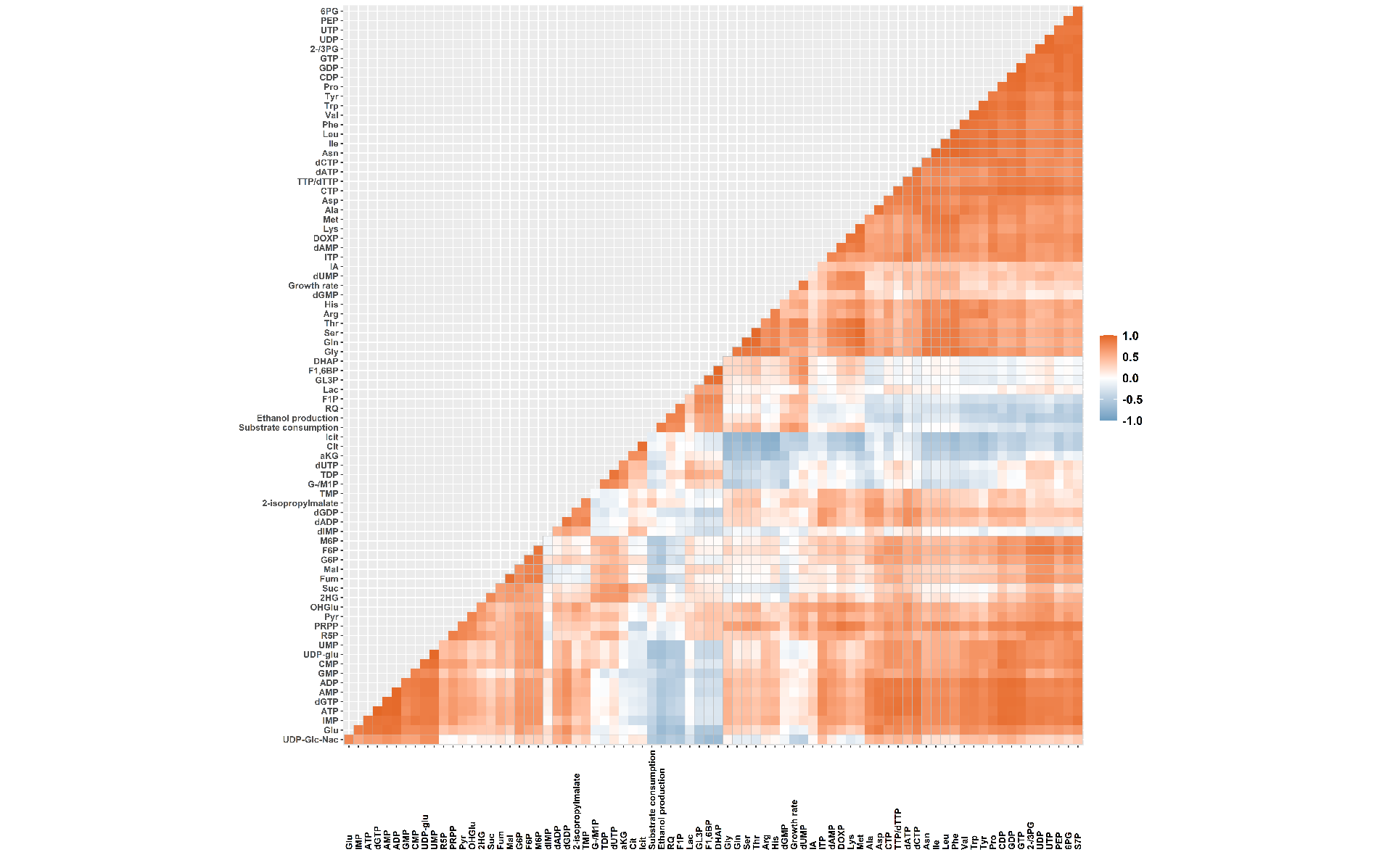


**Additional file 2: Figure S5.** Spearman rank correlation of combined exo and endo metabolite data for all cultivation conditions, except batch stationary phase.


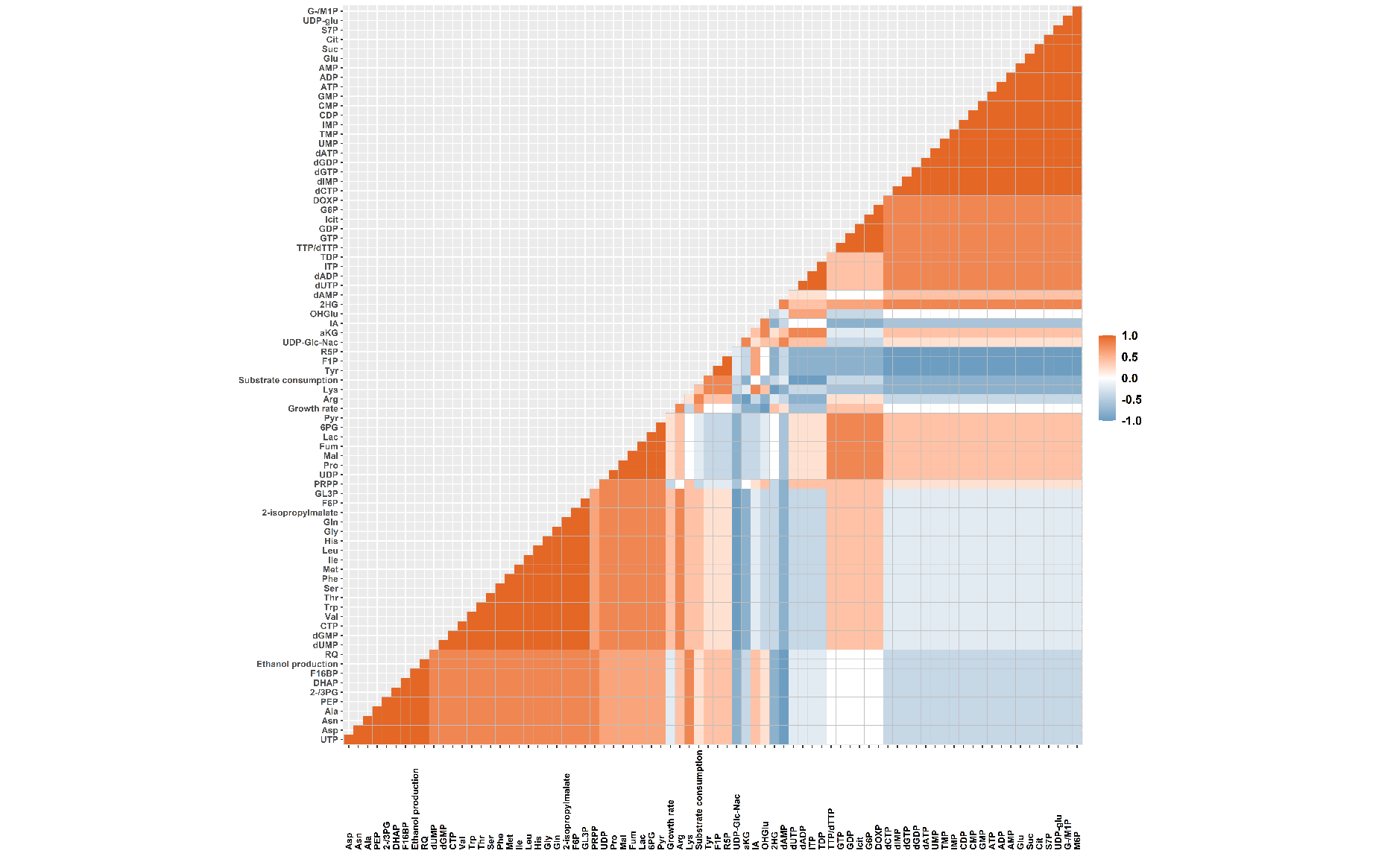


**Additional file 2: Figure S6.** Spearman rank correlation of combined exo and endo metabolite data for the four batch growth phase conditions.


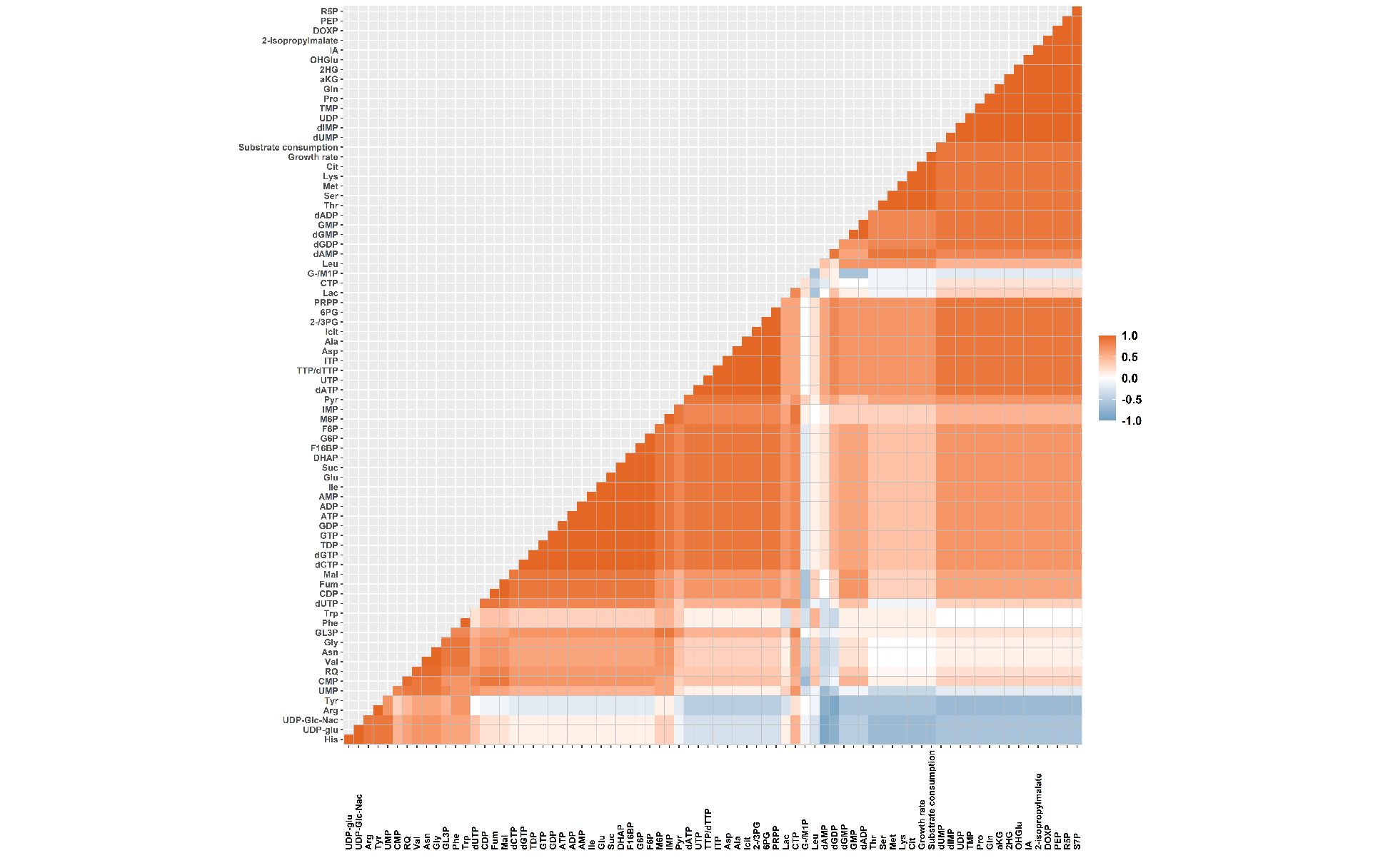


**Additional file 2: Figure S7.** Spearman rank correlation of combined exo and endo metabolite data for the Low Glucose limited chemostats.


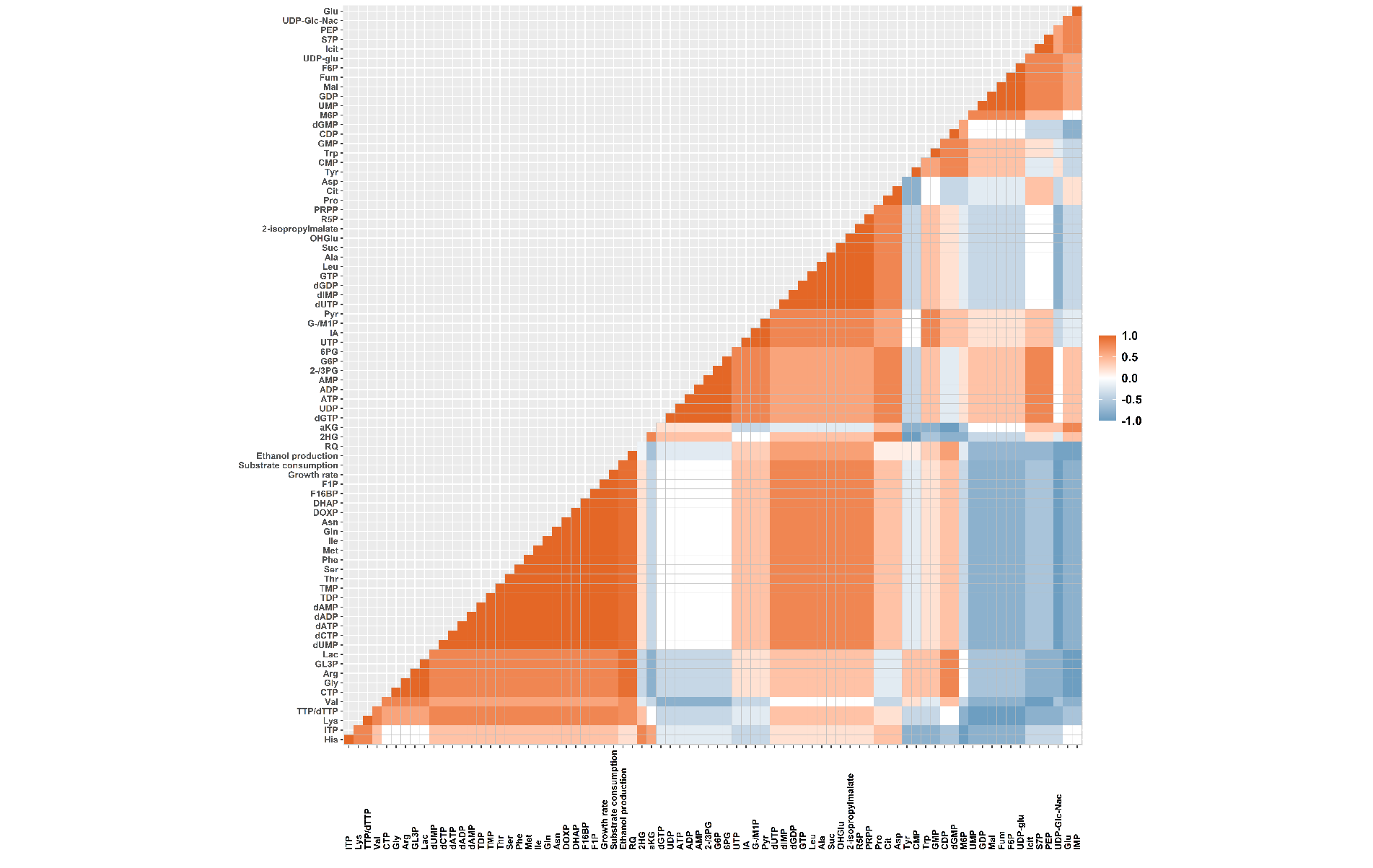


**Additional file 2: Figure S8.** Spearman rank correlation of combined exo and endo metabolite data for the High Glucose limited chemostats.


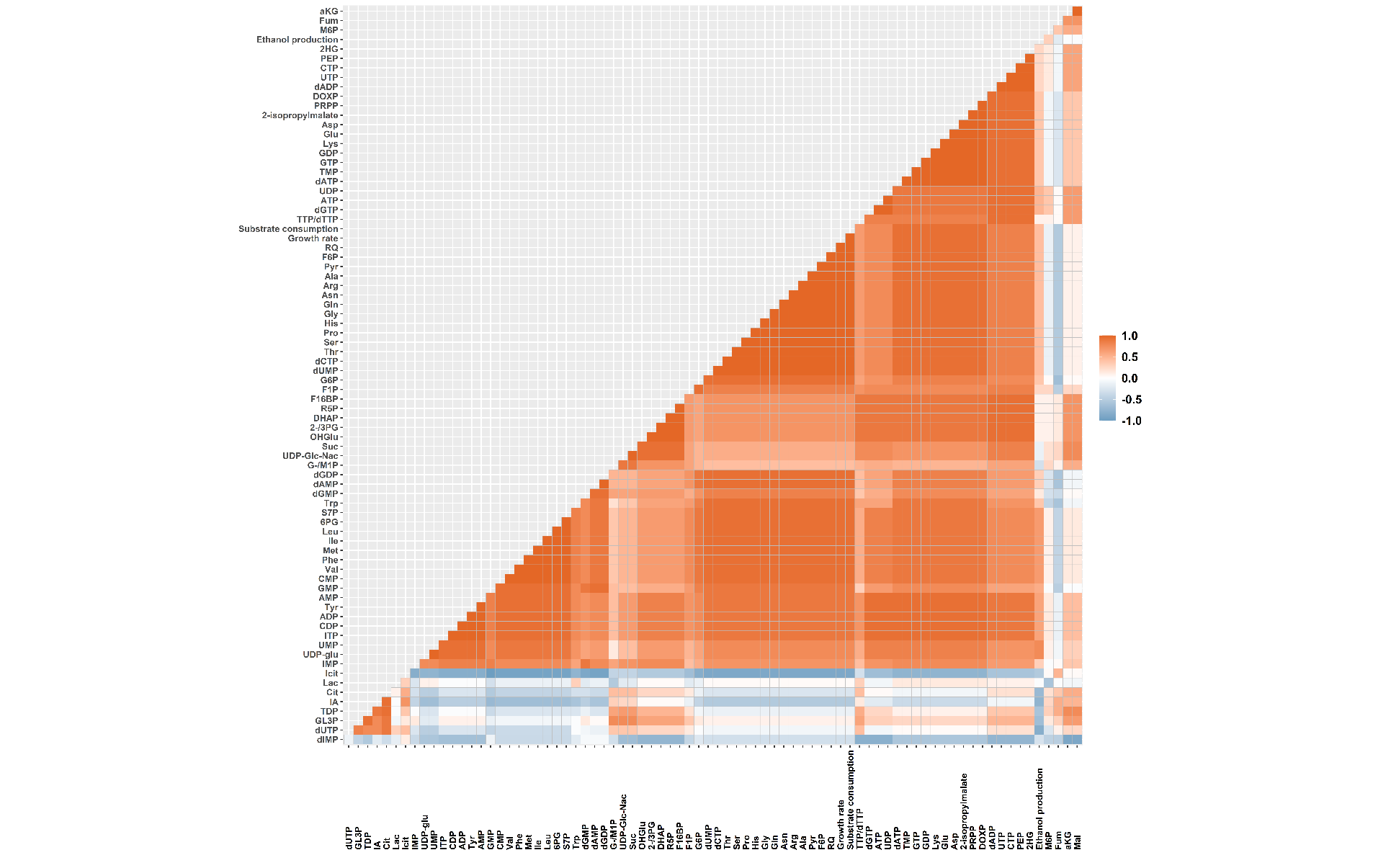


**Additional file 2: Figure S9.** Spearman rank correlation of combined exo and endo metabolite data for the Nitrogen limited chemostats.


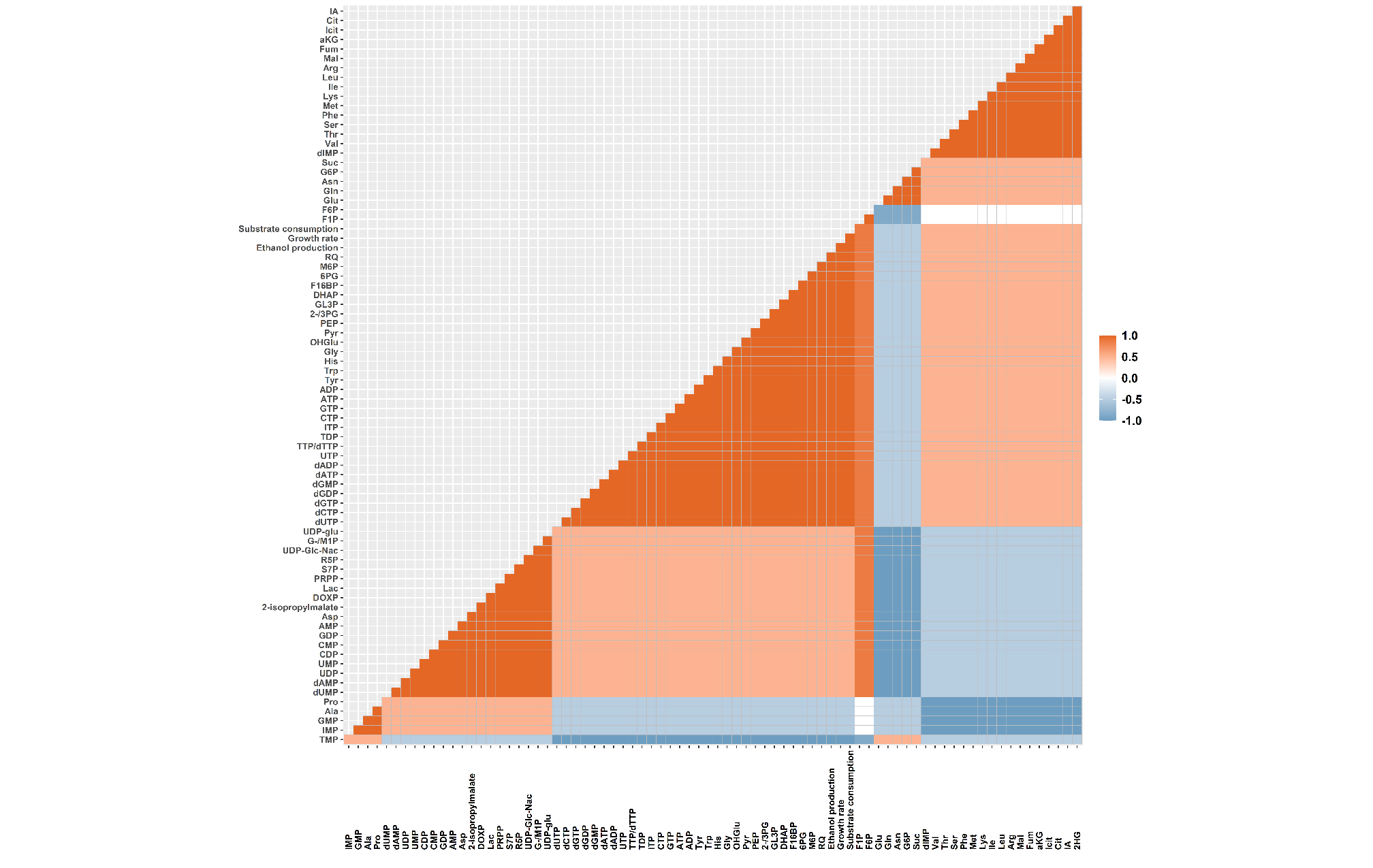


**Additional file 2: Figure S10.** Spearman rank correlation of combined exo and endo metabolite data for the Phosphate limited chemostats.


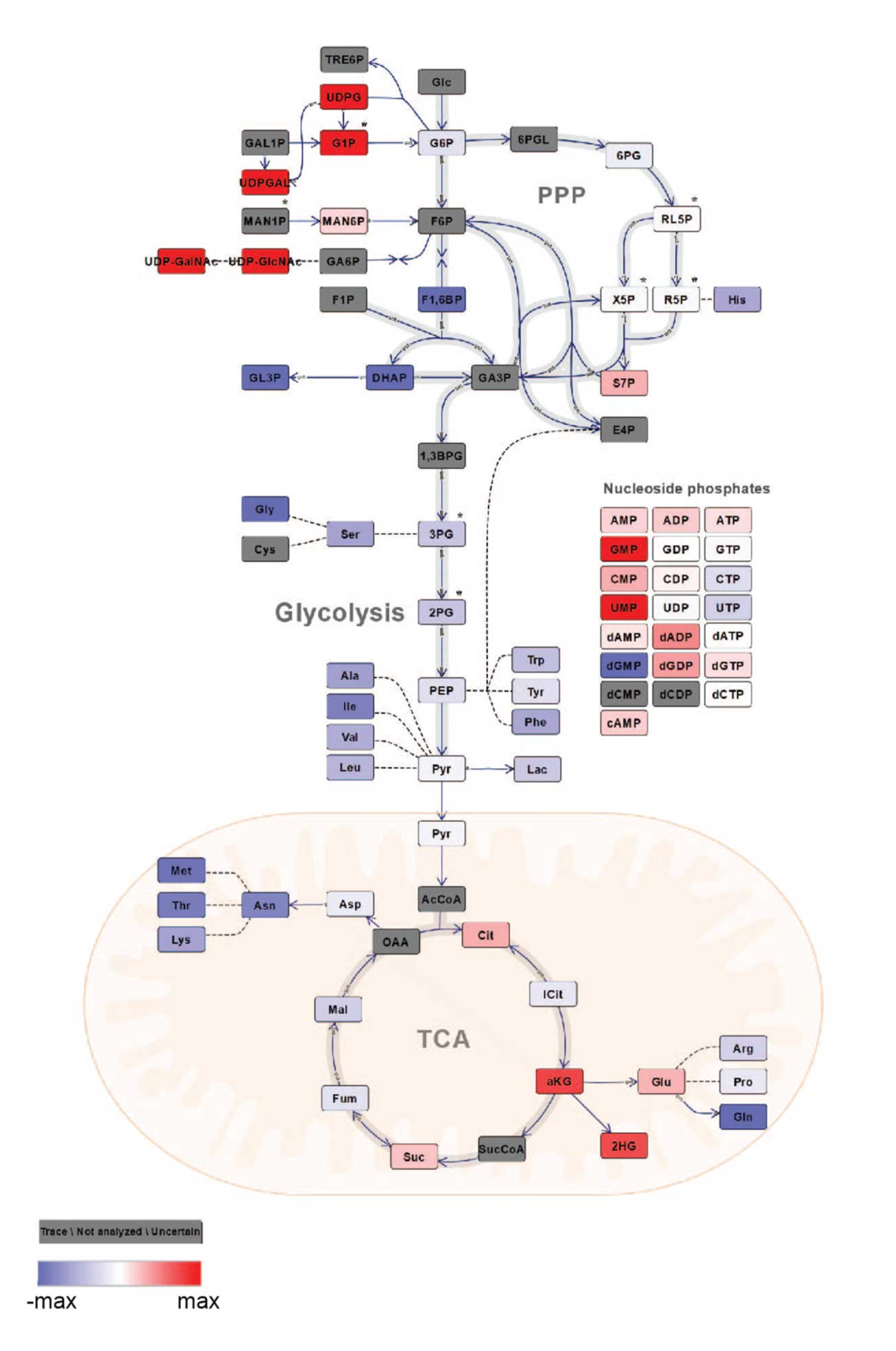


**Additional file 2: Figure S11.** A plot showing changes in the metabolites of central metabolic pathways. This plot is plotted based on logarithmic value (base 2) of ratio of intracellular metabolites concentration of yeast cultivated on galactose to yeast cultivated on glucose’ in exponential phase of batch operation. GAL1P is highly expressed in galactose but not detected in glucose grown yeast in batch reasoning gray color in plot.


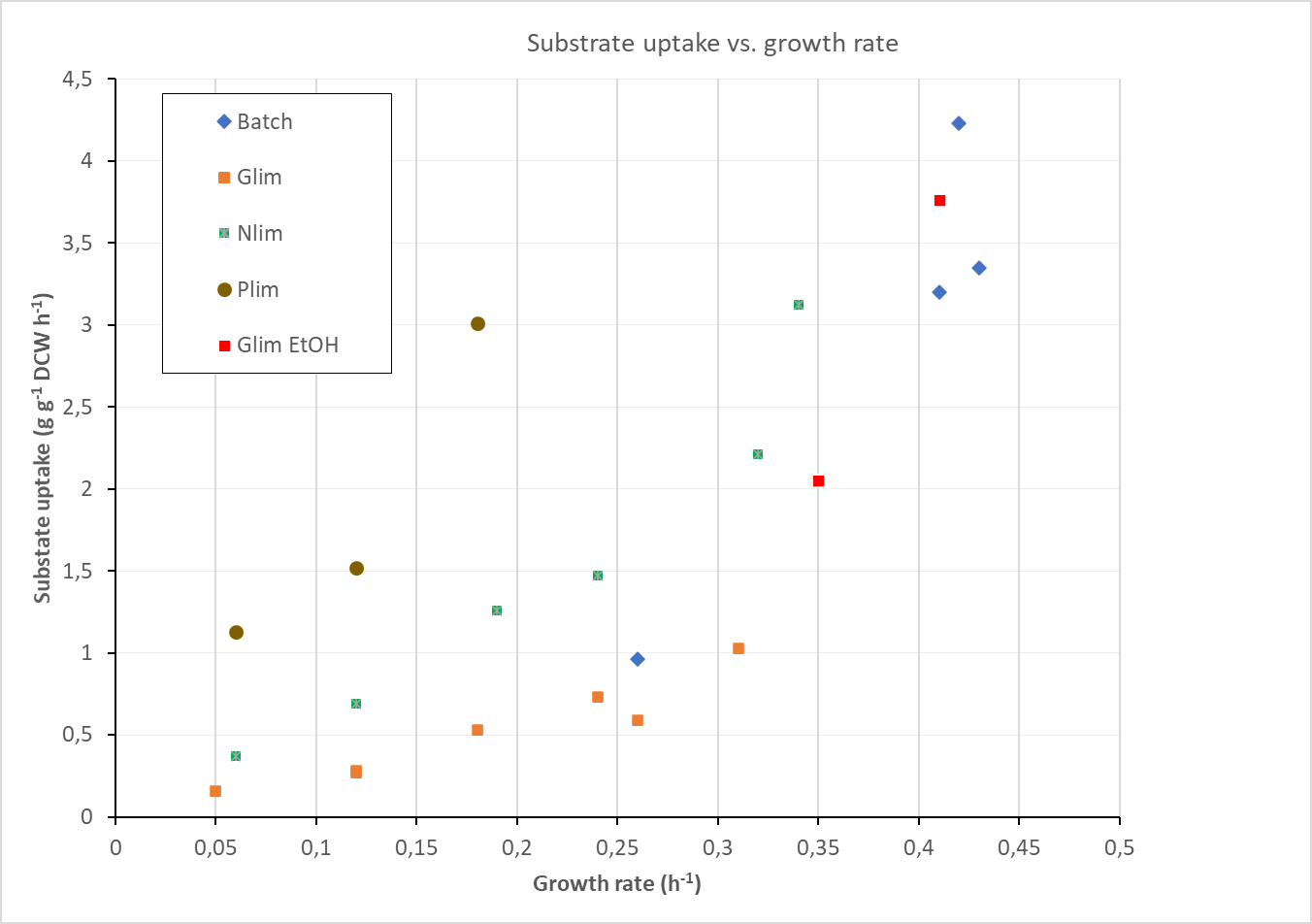


**Additional file 2: Figure S12.** Substrate consumption vs. growth rate for all cultivation conditions. Glim is divided into two categories, those with no ethanol production and the two at highest dilution, i.e growth rate, where ethanol production were detected.
